# Correlation between cellular uptake and cytotoxicity of polystyrene micro/nanoplastics in HeLa cells: A size-dependent matter

**DOI:** 10.1371/journal.pone.0289473

**Published:** 2023-08-08

**Authors:** Yiming Ruan, Zheng Zhong, Xin Liu, Ziwei Li, Junxian Li, Lili Sun, Hou Sen

**Affiliations:** 1 Guangdong Key Laboratory of Environmental Pollution and Health, School of Environment, Key Laboratory of Philosophy and Social Science in Guangdong Province of Community of Life for Man and Nature, Jinan University, Guangzhou, China; 2 Guangzhou Inspection Testing and Certification Group Co., Ltd., China; 3 CAS Key Laboratory of Soil Environment and Pollution Remediation, Institute of Soil Science, Chinese Academy of Sciences, Nanjing, China; 4 Shandong Huapu Testing Technology Co., Ltd., Yantai, China; King Abdulaziz University, SAUDI ARABIA

## Abstract

The cytotoxicity of micro/nanoplastics (MNPs) is known to be strongly influenced by particle size, but the mechanism is not clear so far. We reported the ability of polystyrene MNPs to be internalized by HeLa cells could be a reason for the size dependent cytotoxicity of MNPs. We found that small MNPs (10 nm and 15 nm in radius) could be efficiently internalized by HeLa cells, MNPs of 25 nm in radius could be slightly internalized by the cells, and larger MNPs could not enter the cells at all. We showed that only MNPs, which could be internalized by cells, had a toxic effect on cell activity in a dose-dependent manner. In contrast, MNPs, which could not be internalized by cells, showed no cytotoxicity even if at extremely high concentrations. We attributed the correlation between the size-dependent uptake of MNPs and the size-dependent cytotoxicity of MNPs to the enhanced reactive oxygen species (ROS) level and abnormal gene expression. Our study pointed out that cellular uptake is one of the most fundamental mechanisms for the cytotoxicity of MNPs.

## Introduction

In recent years, MNPs have gradually raised intensive concerns due to their adverse impacts on ecological systems and human health [1–3]. The exposure of MNPs with cells can reduce cell viability [2, 3] and metabolic activity [3], enhance ROS level [4], and trigger inflammatory responses [1]. It was suspected that the size of the MNPs played a key role in its cytotoxicity, since researchers gradually realized that there might be a correlation between the size of MNPs and cytotoxicity [5, 6]. But it is not as simple as the smaller the particle size, the greater the cytotoxicity. For example, Xu et al. found that nanoplastics with smaller particle size could cause stronger cytotoxicity than larger one in human alveolar epithelial cells [7]. In contrast, T. Nomura et al. found that MNPs with large particle size had stronger cytotoxicity than MNPs with smaller particle size in yeast [8]. What’s more, L. Lei et al. found that MNPs with middle size had the strongest toxicity, and increasing or reducing the particle size of MNPs reduced their toxicity in nematodes and zebrafish [9]. In order to understand why MNPs with different sizes have different cytotoxicity, it is necessary to figure out how MNPs affects the cytotoxicity. It was found that sometimes a slight change in the particles size of MNPs could cause a huge change in their cytotoxicity. However, the mechanisms of the size-dependent cytotoxicity of MNPs were still unclear.

MNPs, as a recognized global pollutant, are proved to trigger adverse intracellular effects [10, 11]. For instance, Yan et al. found that the exposure of MNPs induced excess ROS in gastric cancer cells, resulting in a significant decrease in cell viability and apparent cell death [12]. In addition, smaller size MNPs induced more serious adverse effects than larger size MNPs. Studies also attributed the cytotoxicity of MNPs to abnormal gene expression [13, 14]. Xu et al. found that MNPs caused abnormal expression of genes related to the release of cellular proinflammatory cytokines in epithelial cells, and the normal physiological functions of cells were significantly affected [7]. Although it seems that the story of size-dependent cytotoxicity of MNPs can be well ended by the ROS production and gene expression, it is still unknown why MNPs of different sizes could cause different ROS level and gene expression. The key link is missing.

Particles with significant cellular uptake will have a greater chance to contact with intracellular components and thus be more likely to induce the cytotoxic phenomena [15]. It is very likely that cellular uptake played a key role in the size-dependent cytotoxicity of MNPs. Cellular uptake of particles can be divided into two main ways: passive diffusion and active transport [16]. Passive diffusion allows particles to enter the cell directly driven by a difference in concentrations or potentials. Passive diffusion usually occurs during cellular uptake of small particles [17], which may form direct contact with intracellular components and pose a significant cytotoxic threat [15]. Active transport involves caveolae and clathrin mediated endocytosis, which usually occurs during cellular uptake of relatively large particles. Particles that enter the cell in this way form aggregates that are wrapped by membranes and are transported into cells [18, 19]. We suspected that the size-dependent cellular uptake of MNPs were related to the size-dependent cytotoxicity of MNPs. Unfortunately, data is still lacking to confirm the assumption.

The aim of this study is to make up the missing link between the size and the cytotoxicity of MNPs. The uptake of MNPs with different sizes (10, 15, 25, 40, 50, 500 nm in radius) were examined in HeLa cells. Unlike cells that have endocytosis function, where the particles of different sizes can be transported into cells in one big endosome, transportation of particles into HeLa cells need to pass the membrane and is more sensitive to their size. Cell viability, cell death rate, intracellular ROS level, and gene expression induced by MNPs were examined. We explained the size-dependent cytotoxicity of MNPs by their size-dependent cellular uptake.

## Materials and methods

### Reagents

Polystyrene MNPs (non-fluorescence and fluorescence) were purchased from Huge Biotechnology (Shanghai, China). The radius of MNPs were 10, 15, 25, 40, 50 and 500 nm, respectively. Sodium dodecyl sulfate (SDS), Dimethyl sulfoxide (DMSO), NaCl, Na_2_HPO_4_, KH_2_PO_4_ and KCl were purchased from Aladdin (Shanghai, China). Propidium iodide (PI), Hoechst 33342 and 4′,6-diamidino-2-phenylindole (DAPI) was purchased from Solarbio (Beijing, China). 2′, 7′-dichlorofluorescein diacetate (DCFH-DA) was purchased from Sigma Aldrich (Saint Louis, USA). DiD perchlorate was purchased from Coolaber Science & Technology Co., Ltd (Beijing, China). Cell Counting Kit-8 (CCK-8) was purchased from Dojindo (Shanghai, China). Dulbecco′s Modified Eagle Medium (DMEM) and Penicillin-Streptomycin were purchased from Gibco (NY, USA). Fetal bovine serum (FBS) was purchased from Hyclone (South America). Trypsin (bovine pancreas) was purchased from Yuanye (Shanghai, China).

### MNPs characterization analysis

1 mL of 50 mg/mL NPs of different size were dried on a cover glass to produce MNPs powder. MNPs powder and KBr were mixed and ground by a ratio of 1:100 for tablet pressing. The infrared radiation spectra of samples were measured by Fourier-transform infrared spectroscopy (FTIR, IRTracer-100, SHIMADZU, Japan).

Prior to TEM sample preparation, MNPs stock solution was first sonicated (50 Hz and 250 W bath at 25 °C) for 10 min using an ultrasonic cleaner (DK-S11, ZHONGYIGUOKE, China). MNPs were diluted with ultra-pure water to reach a final concentration of 100 mg/L. 5 μL of MNPs was dripped onto the surface of a copper mesh, and the copper mesh was air dried naturally. The sample on the copper mesh was observed with a transmission electron microscopy (TEM, PHILIPS TECNAI 10, Philips, Netherlands) operated at 120 kV.

MNPs were solved in PBS, serum-free medium and culture medium, respectively. The concentration of MNPs solutions was 10 mg/L. The solutions were filtered by a syringe filter with pores of 0.22 μm (PES, JINTENG, Tianjin, China) in diameter before use. The hydrodynamic radius of MNPs was measured with a Malvern Zetasizer Nano (Nano ZS90, Malvern Panalytical, UK) with temperature of 25 °C and scattering angle of 173°.

### Cell culture and exposure of MNPs

HeLa cells were cultured in culture medium, which was prepared with DMEM supplied with 10% fetal bovine serum (FBS) and 1% antibiotic (100 U/mL penicillin and 100 μg/mL streptomycin). MNPs exposure experiments were performed in serum-free medium (DMEM supplied with 1% antibiotic). Cells were maintained in constant temperature cell incubator with 5% CO_2_ at 37 °C.

HeLa cells were subjected to trypsinize, centrifuge and resuspend for collecting cell suspension. The suspension was diluted to a final concentration of 1×10^5^ cells/mL with culture medium (DMEM supplied with 10% FBS and 1% antibiotic) before cultured in 12-well or 96-well plates. Cells were cultured at least for 24 h to achieve a state of completely adherent before carried out for experiments.

### Cell viability assay

The previous culture medium in the 96-well plates was removed, and cells was washed once with phosphate buffered saline (PBS) to get rid of residual serum proteins. Then MNPs solutions were mixed with serum-free medium at a ratio (v/v) of 1:100 to concentrations of 0, 1, 10, 20, 40, 80, 100, 200 and 500 mg/L. Then 200 μL serum-free medium containing MNPs was added to indicated well with three parallels. After being incubated for 4 hours at 37 °C, cells were washed twice with PBS. CCK-8 solutions were diluted with serum-free medium at a ratio (v/v) of 1:10. Then 100 μL serum-free medium containing CCK-8 was added, incubating for 30 minutes at 37 °C. After which, a multifunctional microplate reader (Synergy H1, BioTek, USA) was conducted to measure absorption wavelength of 450 nm optical density. The cell viability was calculated by Eq (1).

$$\text{Cell}\;\text{viability}\left(\%\right)=\frac{{\text{OD}}_{\text{t}}{\text{-OD}}_{0}}{{\text{OD}}_{\text{c}}{\text{-OD}}_{0}}\times 100$$ (1)

where OD_0_ was the value of OD_450_ of the serum-free medium containing CCK-8, OD_c_ was the value of OD_450_ of the serum-free medium containing CCK-8 and non-treated cells, and OD_t_ was the value of OD_450_ of the serum-free medium containing CCK-8 and cell samples treated with MNPs. All experiments were repeated for three times.

### Cell death rate assay

Prior to carry out experiments, cells were washed twice with PBS. Then MNPs solutions were mixed with serum-free medium at a ratio (v/v) of 1:100 to concentrations of 0, 1, 10, 20, 40, 80, 100, 200, 500 mg/L. Then 200 μL serum-free medium containing MNPs was added with three parallels. After being incubated for 4 hours at 37 °C, cells were washed twice with PBS. PI and Hoechst 33342 solutions were diluted with serum-free medium at a ratio (v/v) of 1:100. Next, 200 uL serum-free medium containing PI and Hoechst 33342 was added, incubating for 30 minutes at 37 °C. Cells were washed twice with PBS before observation using fluorescence microscope. Three images, which were containing red and blue fluorescence channels, were picked up randomly in one well. Imagine J (version 2017) software was used to count the number of spotted cell nucleuses. Blue spotted cell nucleuses represented a cell, while those of red spotted represented a dead cell. The cell death rate was calculated by Eq (2).

$$\text{Cell}\;\text{death}\;\text{rate}\;(\%)=\frac{{\text{N}}_{\text{r}}}{{\text{N}}_{\text{b}}}\times 100$$ (2)

where N_r_ was number of red spotted nucleuses within a field of view and N_b_ was number of blue spotted nucleuses within a field of view.

### Reactive oxygen species (ROS) measurement

Cells were washed twice with PBS. MNPs solutions were mixed with serum-free medium at a ratio (v/v) of 1:100 to the concentrations of 0, 1, 10, 20, 40, 80, 100, 200, and 500 mg/L, respectively. Then 200 μL serum-free medium containing MNPs was added with three parallels. After being incubated for 1 h at 37 °C, cells were washed twice with PBS. DCFH-DA solution was diluted with serum-free medium at a ratio (v/v) of 1:1000 to a concentration of 10 μM. 200 μL of serum-free medium containing DCFH-DA was added, incubating for 30 minutes at 37 °C. The cells were wash once with PBS, and 50 μL of PBS was added in order to prevent cells from drying. A multifunctional microplate reader was conducted to measure the intracellular fluorescence intensity at an excitation wavelength of 488 nm and an emission wavelength of 525 nm.

### Fluorescence microscope observation and images processing

Fluorescent and non-fluorescent MNPs in the same sizes were mixed at a ratio (v/v) of 1:9, and then diluted with serum-free medium to concentrations of 100 mg/L. 2 mL of serum-free medium containing the mixed MNPs were added. After incubated for 1 h, cells were washed three times with PBS. A EVOS FLoid^®^ Cell Imaging Station (Life Technology, USA) was used for obtaining bright field and green fluorescent channel images, respectively. Photoshop software (Adobe Photoshop 2020) was conducted to merge bright field and green fluorescent channel images.

### Confocal Laser Scanning Microscopy (CLSM) measurement

HeLa cells were seeded at 1×10^5^ cells/mL in confocal dish and cultured for 12 h. After that, the culture medium was removed and cells were washed three times with PBS. DiD solution was diluted with serum-free medium at a ratio (v/v) of 1:100 to a concentration of 5 μM. Then, 1 mL of serum-free medium containing DiD was added to the dish. After cultured for 30 minutes, cells were wash three times with PBS before exposed to fluorescent MNPs of indicated sizes. The mixed MNPs of 10, 15, 25, 40, 50 and 500 nm in radius were diluted with serum-free medium to a concentration of 100 mg/L. 1 mL of serum-free medium containing mixed MNPs was added to the dish. After being incubated for 1 h, cells were washed with PBS three times to get rid of residual mixed MNPs. 1 mL of serum-free medium was added to the dish in case of cells drying during observation. Cells were observed with a confocal microscopy (LSM800, Zeiss, Germen). All images acquisition were performed with ×63 oil-immersion objective. The excitation wavelength of confocal laser was 646 nm, and the emission wavelength was 663 nm for DiD perchlorate. The excitation wavelength was 488 nm, and the emission wavelength was 525 nm for fluorescent MNPs. Green and red channels of cells were obtained respectively. Image processing program (ZEN Software) overlaid the images automatically, outputting merged images.

### Real-time quantitative PCR analysis

The cells were seeded at 1×10^5^ cells/well in 12-well plate. After cell culture for 24 h, the cells were treated with 100 mg/L MNPs of 10, 15, 25, 40, 50 and 500 nm in radius, respectively. After 4 h exposure, cells were detached by mechanical dissociation and subjected to gene expression analysis. The expression of marker genes was determined using real-time quantitative PCR (RT-qPCR) as follows. Total cellular RNA was extracted from exposed cells using a trizol Kit according to the manufacturer’s instructions. 1 μg of total RNA was reverse-transcribed into cDNA using the Evo M-MLV RT Mix Kit according to the manufacturer’s protocol. A standard reaction was prepared in 96-well plates (Bio-Rad, USA) for RT-qPCR. The reaction mixture was composed of 10 μL of 2X SYBR^®^ Green Pro Taq HS Premix, 0.4 μL of the forward and reverse primers (10 μM), 1 μL of cDNA, 0.4 μL of ROX Reference Dye (4 μM), and distilled water to a final volume of 20 μL. The thermocycling conditions were 95 °C for 30 s, followed by 40 cycles of 95 °C for 5 s and 60 °C for 30 s. Normalization of the data was performed using the housekeeping gene GADPH as an endogenous control. RT-qPCR was performed using a fluorescence quantitative PCR instrument (CFX96 Touch, Bio-Rad, USA). The expression of GAPDH, Bax, Bcl-2, SOD1, CAT, Fas and FADD genes were measured. The purity and integrity of RNA were determined by the A260/A280 and A260/A230 value (Table S1 in S1 File). The primer sequence form primer bank is in Table S2 in S1 File. All measurements were repeated for three times.

### Statistical analysis

All experiments were repeated at least three times. The results were presented as mean ± standard deviation. Data was analyzed using SPSS 25.0 (IBM SPSS Statistic 25) software. Graphs were performed using Origin (OriginLab, Origin 2018) software. Significant differences were assessed using independent-samples t-test. Statistically significant difference was defined as * P < 0.05, ** P < 0.01, and *** P < 0.001. A value of P < 0.05 was considered to be significant, a value of P < 0.01 was considered to be very significant and a value of P < 0.001 was considered to be extremely significant.

## Results and discussion

### Characterization of MNPs

**Fig 1:**
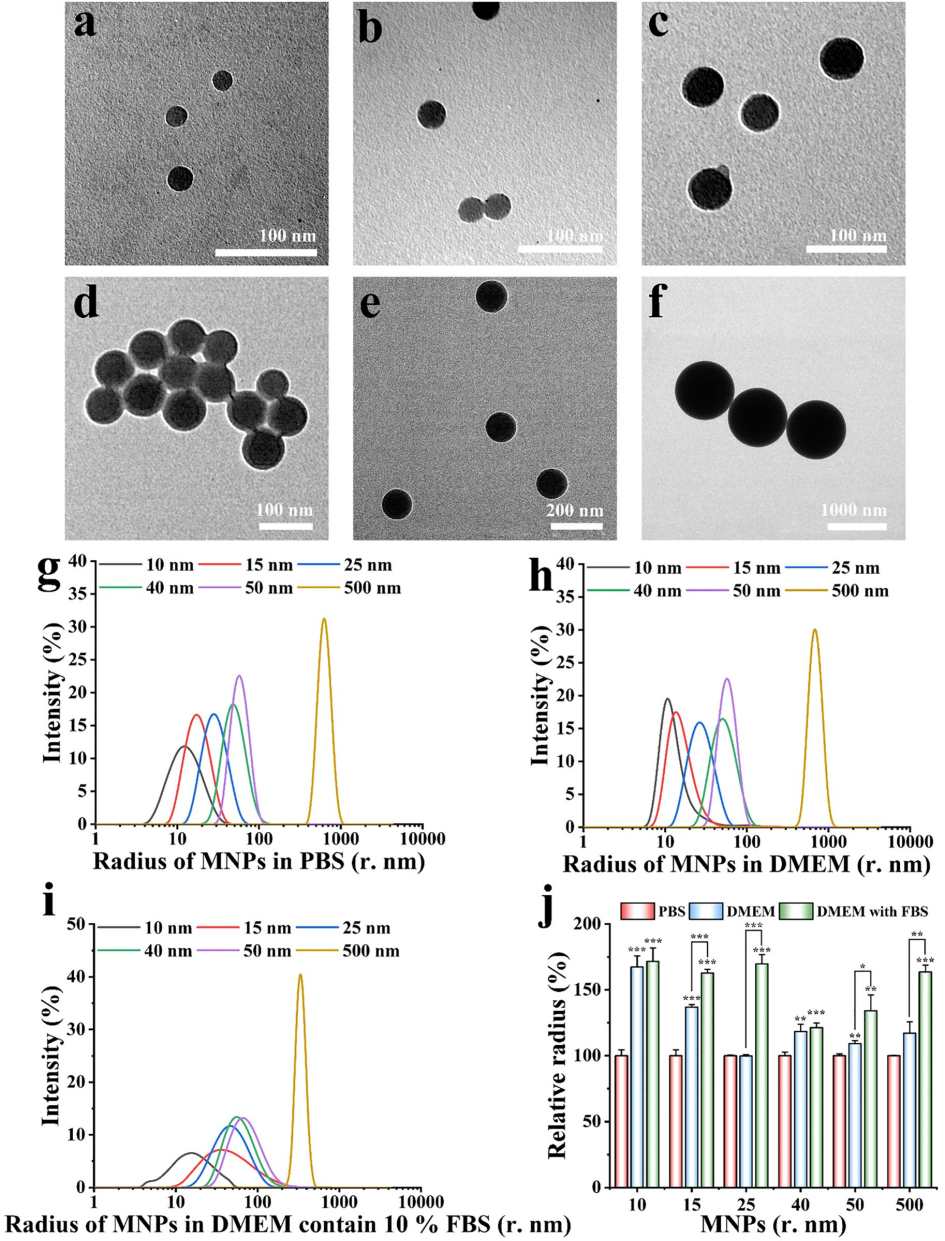
Characterization of MNPs. TEM image of MNPs of (a) 10 nm, (b) 15 nm, (c) 25 nm, (d) 40 nm, (e) 50 nm, and (f) 500 nm in radius, respectively. The scale bar in (a), (b), (c), and (d) is 100 nm. The scale bar in (e) and (f) is 200, and 1000 nm, respectively. Radius of MNPs in (g) PBS, (h) DMEM, and (i) culture medium. (j) Relative sizes of NPs in different solutions compared to those in PBS. Significant differences between and within groups were tested with independent-samples t-test (* represents p < 0.05, ** represents p < 0.01, and *** represents p < 0.001).

The chemical structure of MNPs was studied by FITR, where the characteristic peaks were consistent with those of polystyrene (Fig S1 in S1 File). The size of MNPs measured by TEM was consist with their commercial data. We measured the dispersion/agglomeration state of MNPs in PBS, serum-free culture medium, and culture medium with serum (Fig 1). No aggregation was observed in PBS (Fig 1g). MNPs in serum-free medium were a little larger than commercial data (Fig 1h), but the difference was not significant. Size of MNPs in the culture medium was larger than commercial data (Fig 1i). This can be attributed to the adsorption of serum proteins onto MNPs surfaces [20].

### Cell viability and death rate assay

**Fig 2:**
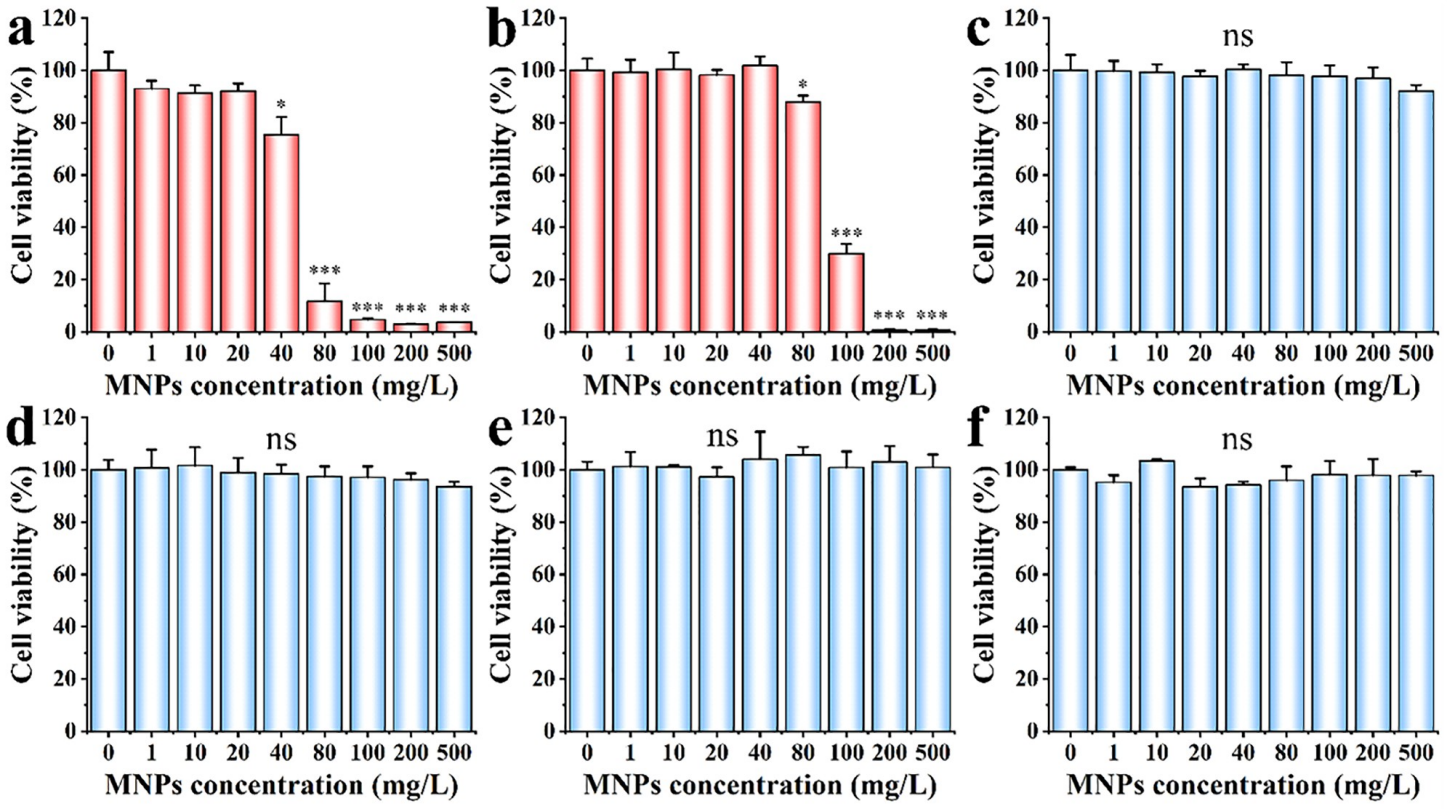
Cytotoxicity of HeLa cells exposed to MNPs of (a) 10 nm, (b) 15 nm, (c) 25 nm, (d) 40 nm, (e) 50 nm and (f) 500 nm in radius. Cells were subjected to 4 h MNPs exposure. Results were represented with mean ± SD. Significant differences between and within groups were tested with independent-samples t-test (* represents p < 0.05, ** represents p < 0.01, and *** represents p < 0.001). Results demonstrate that smaller MNPs that can be internalized by cells had a greater effect on cell viability than those that cannot.

Cell viability and death rate, as common endpoints of cytotoxicity, were performed to evaluate the cytotoxicity under different radius of MNPs to HeLa cells (Fig 2). For MNPs of 10, 15 and 25 nm in radius, significant decrease in cell viability was observed. MNPs of 10 nm in radius caused no significant decrease in cell viability with concentrations of 1, 10, and 20 mg/L compared to the control. Cell viability decreased to ca. 70% when the concentration increased to 40 mg/L. A dramatic decrease (ca. 10%) occurred when the concentration increased to 80 mg/L. Then, cell viability decreased to a minimum (approximately 3%) at a concentration of 100 mg/L (Fig 2a). The IC_50_ of MNPs of 10 nm in radius from concentration-effect curve was 51.2 ± 5.7 mg/L (Fig S2a in S1 File). For MNPs of 15 nm in radius, the cytotoxicity trend was similar to that of MNPs of 10 nm in radius, except the IC_50_ was increased to 93.5 ± 0.5 mg/L (Fig S2b in S1 File). When exposed to MNPs of 25 nm in radius, the cell viability decreased to ca. 90% at a concentration of 500 mg/L. It can be seen from the above data that MNPs with small size have obvious cytotoxicity, and the cytotoxicity was positively correlated to the concentration of MNPs.

In contrast, for MNPs of 40, 50 and 500 nm in radius, no significant decrease in cell viability was observed in the same range of concentrations (Fig 2c–2f). This result was similar to Malugin et al. and Stock et al. that gold nanoparticles of 45 nm and MNPs of 500 nm in radius did not induce obvious decrease in cell viability [21, 22].

It should be noted that the decrease in cell viability was not due to the difference in molar concentrations of MNPs. Given the total amount (by weight) of MNPs was fixed, the smaller MNPs had more particle number. We experimentally showed that the size-dependent toxicity of MNPs was not caused by the difference in particle number. We compared the effects of MNPs of 10 and 50 nm in radius on cell viability at the same molar concentrations (Fig S3 in S1 File). The results showed that MNPs of 10 nm in radius decreased cell viability, but MNPs of 50 nm in radius did not decrease cell viability. Results indicated that MNPs with large size did not induce obvious cytotoxicity even at extremely high concentrations. The number of MNPs was not the main reason for the decrease in cell viability.

**Fig 3:**
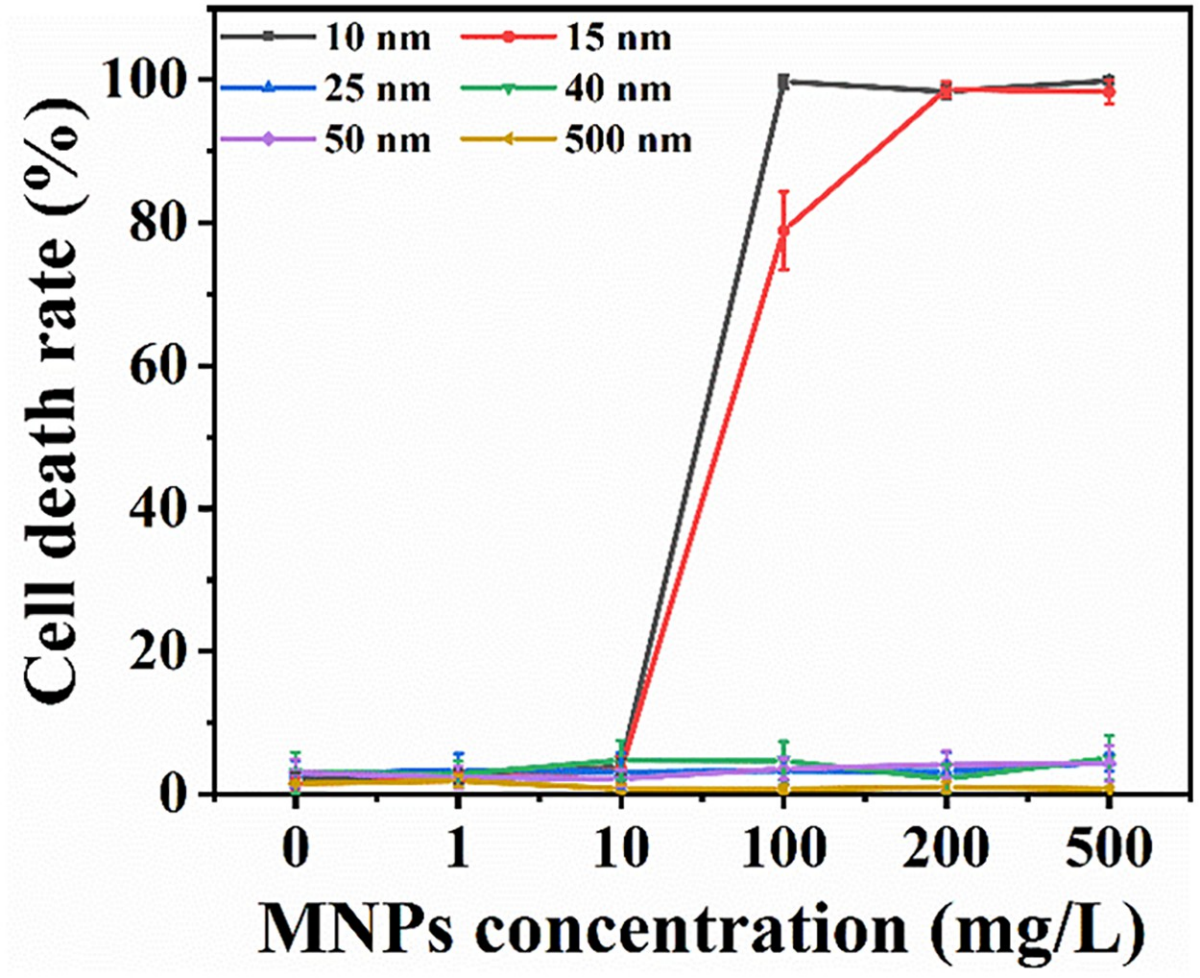
Cell death induce by MNPs with various sizes. Cells were incubated with MNPs for 4 h. The exposure concentration was 100 mg/L. Results were represented with mean ± SD. Results demonstrate that MNPs that can be internalized by cells induce severe cell death, while MNPs that cannot enter cells have no obvious effects on cell death.

We used PI dye for cell death rate detection. Cell death rate can indicate the changes in cell membrane permeability to some extent, because PI dyes cannot stain the nuclei of living cells with intact cell membranes [23, 24]. Results show that MNPs of 10 and 15 nm in radius induced apparent cell death. Cell death rate was nearly 100% at high concentrations, suggesting a dramatic change in cell membrane permeability. This result was similar with Lun et al. that Ag nanoparticles of 12 and 17 nm in radius induced obvious cell death [25]. MNPs of 25, 40, 50 and 500 nm in radius did not induce obvious cell death even at an extremely high concentrations of 500 mg/L (Fig 3). Smaller MNPs induced more significantly cell death than larger size, suggesting that small MNPs may have damaged cell membrane integrity, while large MNPs did not.

### Cellular uptake of MNPs

**Fig 4:**
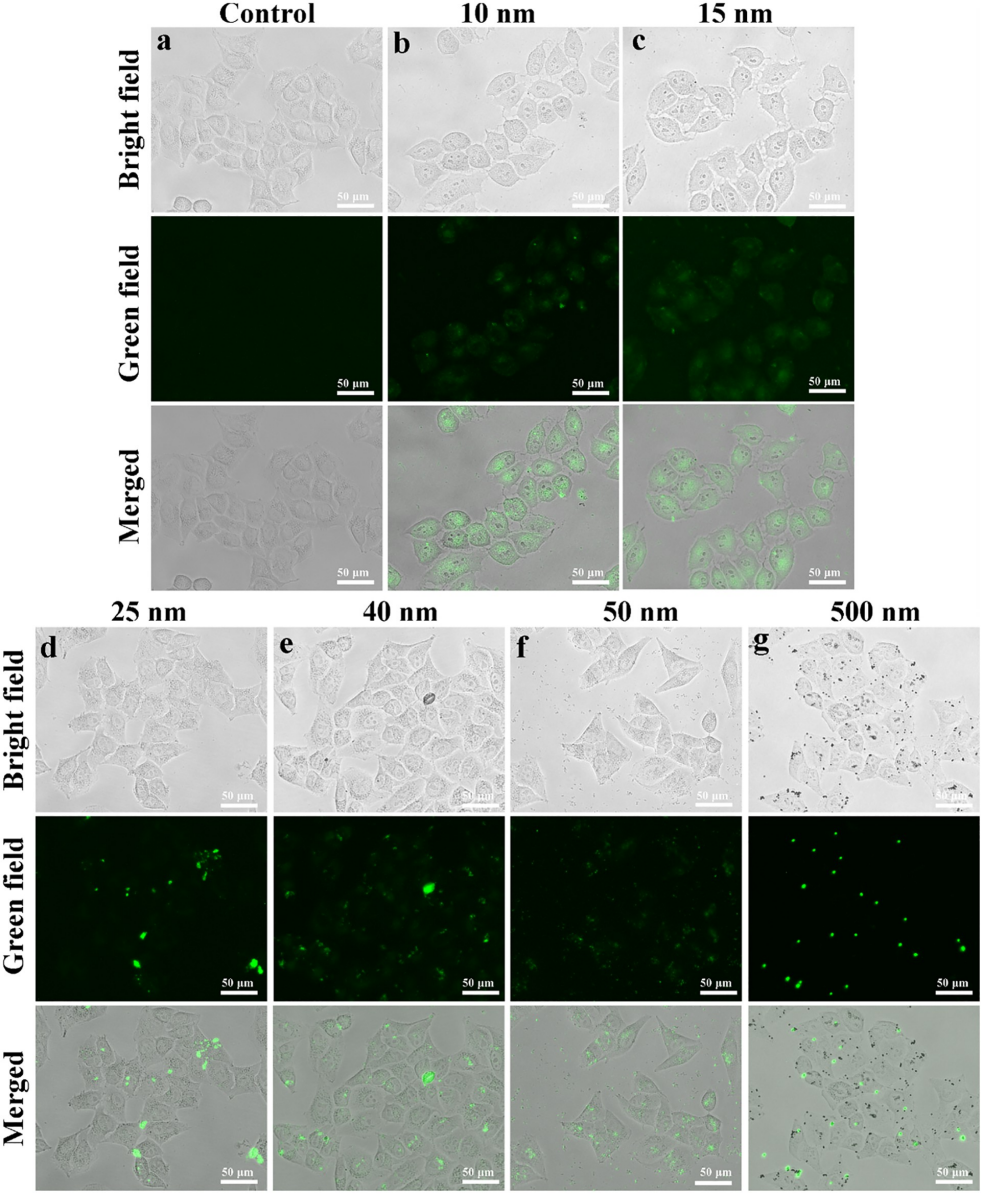
Changes in cell morphology induced by MNPs. The exposure concentration was 100 mg/L. Cells were incubated with MNPs of different sizes: (a) control, (b) 10 nm in radius, (c) 15 nm in radius, (d) 25 nm in radius, (e) 40 nm in radius, (f) 50 nm in radius, (g) 500 nm in radius. The top row is bright field images of cells. The middle row is images of the position of green fluorescence MNPs. The bottom row is the merged images of bright and green field images. The images demonstrate that MNPs with smaller sizes (10, 15 nm in radius) have a more pronounced effects on cell morphology than larger ones (25, 40, 50, 500 nm) compared to the control.

We studied the cellular uptake of MNPs as a function of their particle size. MNPs were labeled with fluorescent dyes and incubated with the cells for 1 h. The extra MNPs were removed and the cell were observed under a fluorescence microscope. With the absence of MNPs, no fluorescence was observed (Fig 4a). With the presence of MNPs of 10 and 15 nm in radius, strong green fluorescence signal was observed inside the cells, indicating that MNPs of these sizes successfully entered the cells. The contour of the cells was irregular compared to the control group, suggesting cell membrane was damaged by MNPs (Fig 4b and 4c). These results were similar with the findings of Sandra et al. that cellular uptake of nanoparticles with sizes of 20 nm in radius was obvious, and the cell morphology was changed [26]. On the contrast, for MNPs of 25, 40, 50, 500 nm in radius, the green fluorescence was relatively less and unevenly distributed in cells (Fig 4d–4g). And the cell morphology did not change significantly. Results suggested that these sizes of MNPs might not have obvious cellular uptake, but mainly adhered on the cell membrane.

**Fig 5:**
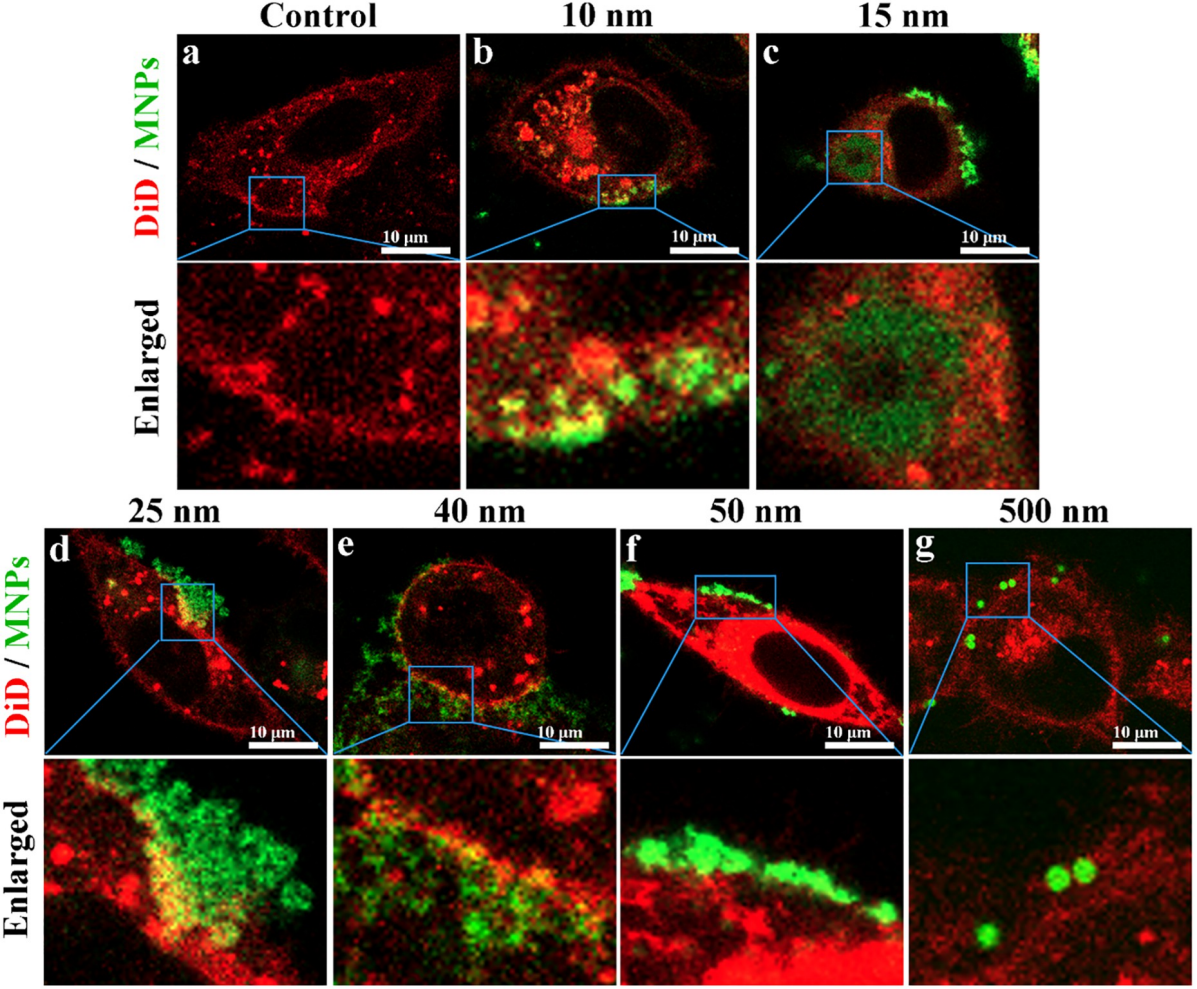
Distribution of MNPs in HeLa cells. The exposure concentration was 100 mg/L. Green regions represent MNPs, and red regions represent cell membrane. Cells were incubated with MNPs of different sizes: (a) control, (b) 10 nm in radius, (c) 15 nm in radius, (d) 25 nm in radius, (e) 40 nm in radius, (f) 50 nm in radius, (g) 500 nm in radius. The top row of images is from the middle of the cells. The bottom row of images is magnified images. The images demonstrate that smaller MNPs such as 10, 15, 25 nm can be internalized, while larger MNPs of 40, 50, 500 nm inradius are primarily on the cell surface.

In order to observe cellular uptake of MNPs and the distribution of MNPs in cells more precisely, CLSM was used for further observation. For MNPs of 10, 15 and 25 nm in radius, green fluorescence MNPs was located in the cytoplasm but not in the nucleus (Fig 5b–5d). Results confirmed that MNPs of 10, 15 and 25 nm in radius have obvious cellular uptake. The result was consistent with the findings of Johnston et al. that MNPs of 10 nm in radius could be internalized by human hepatocyte carcinoma cell and located at cytoplasm [27]. For MNPs of 40, 50 and 500 nm in radius, no obvious green fluorescence was observed inside the cells, but mainly appeared near the cell membrane (Fig 5e–5g). This finding was consistence with Zauner et al. that the uptake of MNPs with a radius larger than 46 nm was almost undetectable in human hepatocyte carcinoma [28].

It can be seen from the above results that the cellular uptake of MNPs by Hela cells basically followed the rule that the smaller the particle size is, the more likely the cellular uptake was. This can be explained by small size of the MNPs facilitates entry into the cell through passive diffusion in response to the concentration difference [29, 30]. MNPs with larger sizes may enter the cell through active transport such as caveolae and clathrin mediated endocytosis [18, 19]. Cellular uptake of MNPs of 10, 15 and 25 nm in radius by HeLa cells is likely to be carried out in the above manners. For those large MNPs, cellular uptake mainly proceeds in phagocytosis way [31], but phagocytosis can only be performed by some cells such as macrophages. Since HeLa cells are not phagocytes, some MNPs with large sizes cannot enter the cells in phagocytosis way [31]. The result was consistent with the findings of Rejman et al. that no significant cellular uptake of MNPs of 500 nm in radius was observed in non-phagocytes [19].

### ROS level under different cellular uptake

**Fig 6:**
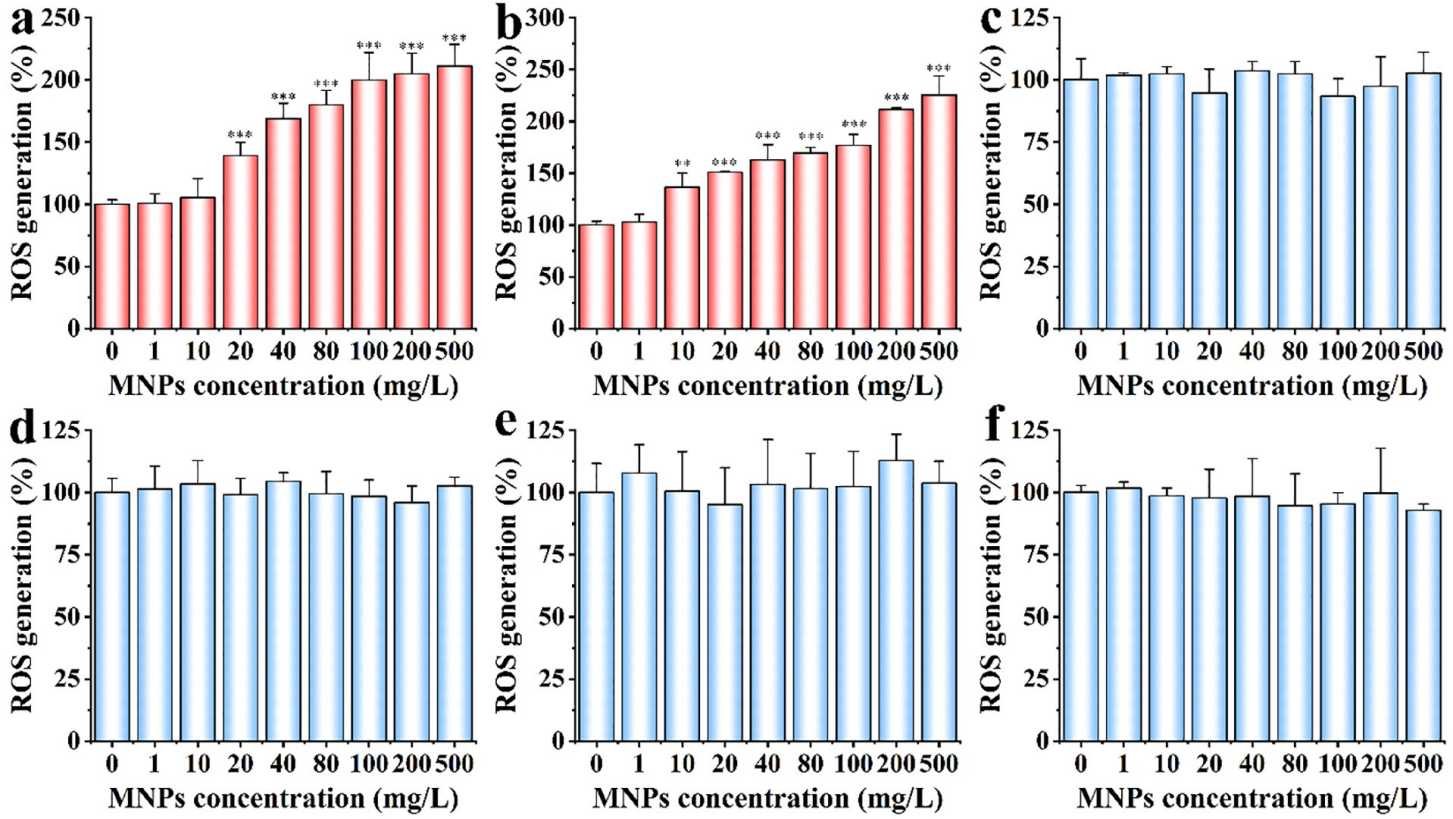
ROS generation of HeLa cells exposed to MNPs of (a) 10 nm, (b) 15 nm, (c) 25 nm, (d) 40 nm, (e) 50 nm and (f) 500 nm in radius. Cells were subjected to 4 h MNPs exposure. Results were represented with mean ± SD. Significant differences between and within groups were tested with independent-samples t-test (* represents p < 0.05, ** represents p < 0.01, and *** represents p < 0.001). Results demonstrate that smaller MNPs that can be internalized by cells induced more cellular ROS generation than those that cannot.

Elevated ROS levels leading to cell death are often cited as one of the mechanisms to explain cytotoxicity [32, 33]. For MNPs of 10 and 15 nm in radius, ROS level gradually increased with the increase of exposure concentrations (Fig 6a and 6b). For MNPs of 25, 40, 50 and 500 nm in radius, there was no obvious changes of ROS level in the range of 0–500 mg/L exposure concentration (Fig 6c–6f). This result well agreed with the data of cell death and cell viability assays (Figs 2 and 3). MNPs with small size can easily penetrate cell membranes and other biological barriers into living organisms and cause cellular dysfunction, which is shown as a rise in ROS production [34, 35]. Excessive ROS levels can induce oxidative stress, resulting in the inability of cells to maintain normal physiological redox regulation [36].

### Real-time quantitative PCR analysis

We studied the change in gene expression with the presence of the uptake-dependent cytotoxicity. We evaluated six genes that were directly related to apoptosis and ROS scavenge: Bax, Bcl-2, SOD1, CAT, Fas, and FADD. The Bax gene (Bcl-2 Associated X-protein) is a pro-apoptotic member of the Bcl-2 gene family. It encodes a 21-kDa protein, named BAX-alpha, which plays a critical role in regulating intrinsic apoptosis [37]. Bcl-2 blocks programmed cell death rather than promoting proliferation [38]. Human SOD1 is responsible for regulating the superoxide levels arising from mitochondrial intermembrane space, cytosol, and peroxisome [39]. CAT is responsible to protect cell against exogenous H_2_O_2_ [40]. FADD is an adapter protein for the apoptotic signaling pathways triggered by Fas-ligand and tumor necrosis factor. Transient FADD overexpression can be sufficient to cause apoptosis in mammalian cells [41].

**Fig 7:**
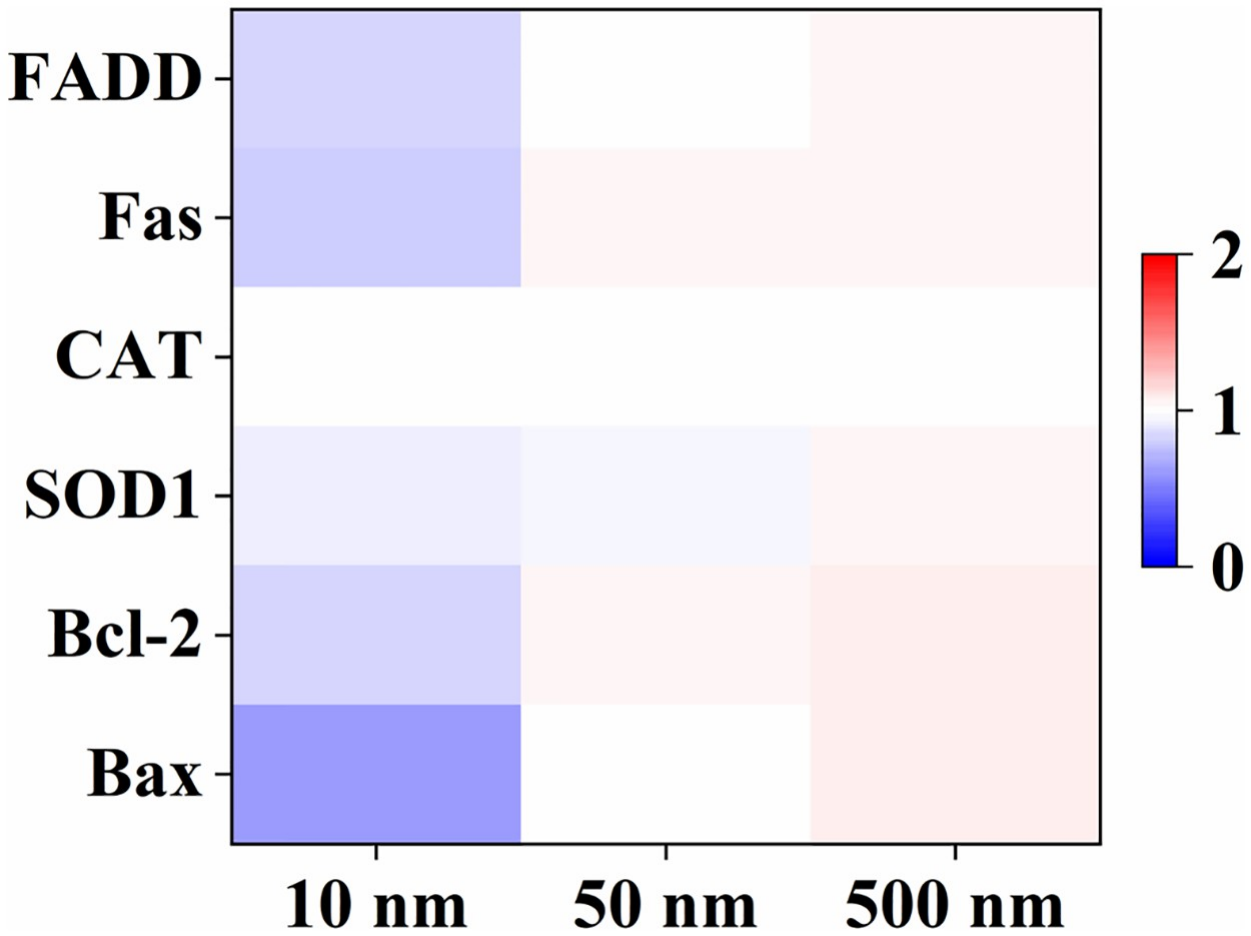
Effects of MNPs of 10, 50, 500 nm in radius with a concentration of 100 mg/L on gene expression levels. The genes related to anti-apoptotic and ROS-protection were regulated with the presence of MNPs.

For MNPs of 10 nm in radius with significant cellular uptake in a concentration of 100 mg/L, all the gene expression decreased (Fig 7). The phenomenon well agreed with the results that MNPs of 10 nm in radius caused a severe cell death and the cell viability decreased by ca. 90% (Fig 2a). The gene related to anti-apoptotic, such as Bcl-2, was down-regulated by 1.29 times. In addition, the expression of ROS-protection genes, such as SOD1 and CAT also decreased (Fig 7). As a result, the exposure of MNPs of 10 nm caused an obvious increase in ROS generation (Fig 6a). For MNPs of 50 and 500 nm in radius without significant cellular uptake in the same concentration, none of the genes changed obviously (Fig 7). The phenomenon also agreed with the results that MNPs of 50 and 500 nm in radius did not cause obvious cytotoxicity (Fig 2e and 2f). These results showed that MNPs with obvious cellular uptake had a significant effect on gene expression, while MNPs without obvious cellular uptake did not.

### Correlation between size-dependent cytotoxicity and cellular uptake of MNPs

Cellular uptake of MNPs might act as a bridge between the size of MNPs and its cytotoxicity. Comparing the data of cellular uptake and cytotoxicity of MNPs, we found that MNPs with significant cellular uptake were cytotoxic, while MNPs without significant cellular uptake were non-cytotoxic. The process of MNPs-induced cytotoxicity should be that the cellular uptake of small MNPs destroyed the integrity of cell membrane and also caused the imbalance of cellular homeostasis. Specifically, the deposition of MNPs in the cytoplasm may result in excessive ROS levels, abnormal gene expression, and eventually cell death [42]. Thus, the cytotoxicity of MNPs with obvious cellular uptake get enhanced dramatically.

**Fig 8:**
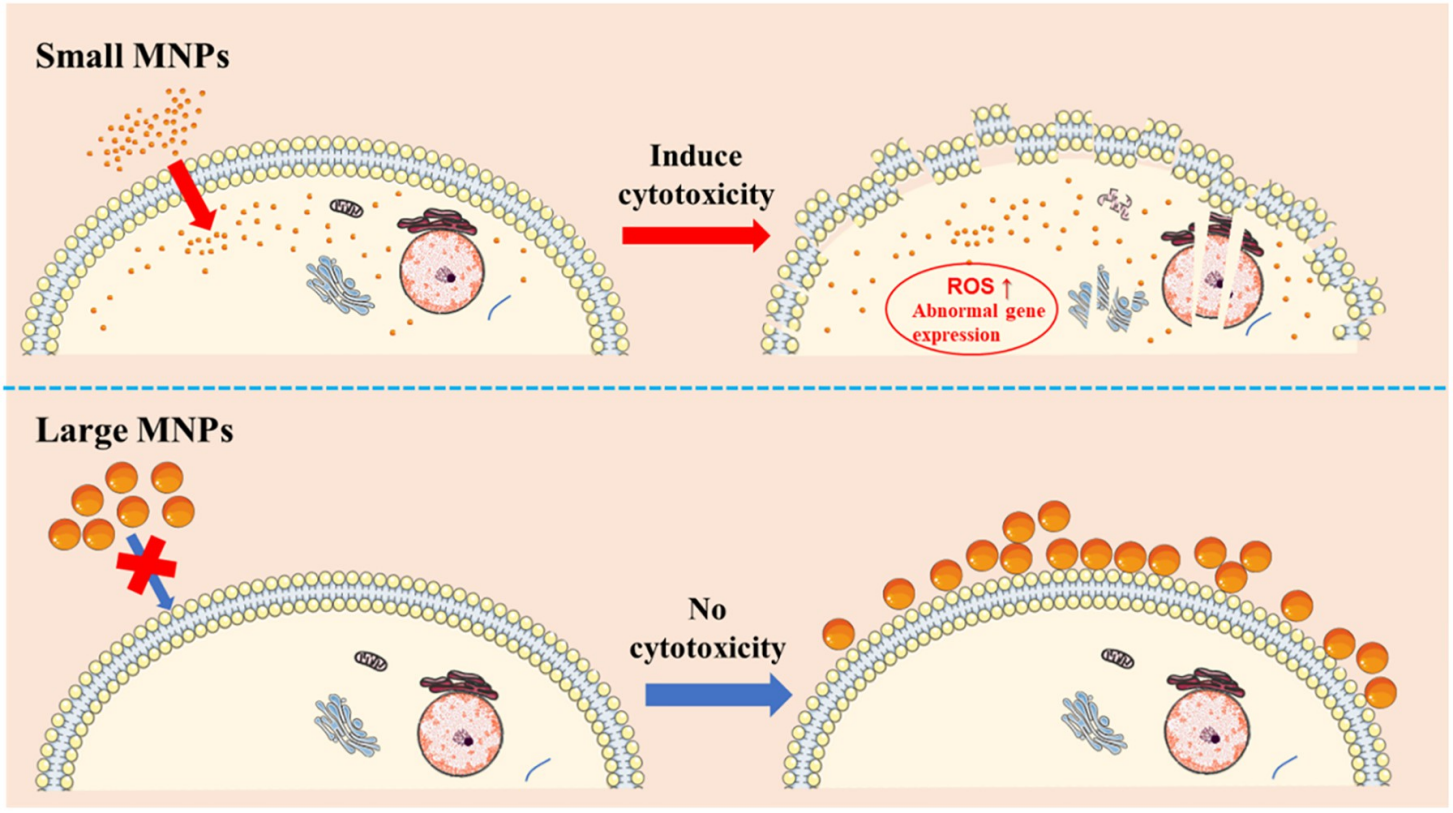
Schematic image showing the correlation between cellular uptake and cytotoxicity, as a function of particle size.

For those MNPs outside the cell or adsorbed on the surface of the cell membrane, they generally did not have significant effects on the cell. We explained the phenomenon by the fact that these MNPs did not have direct contact with the inside organelles of the cell [43, 44]. What’s more, due to the chemical stability of polystyrene MNPs, they did not release monomer to enter the cells, which may be one of the reasons for the non-cytotoxicity of large MNPs [45]. As a result, the size of MNPs not only determines their ability to enter the cells but also determines their cytotoxicity. The theory well explains the size-dependent cytotoxicity of MNPs (Fig 8).

### Implication of this study

Notably, the mechanism of cellular uptake in different cells might be different. For example, a strong involvement of the actin filaments in the uptake process of MNPs in HeLa cells and 1321N1 cells were detected, while microtubules were mostly involved in the A549 cells. Moreover, clathrin-dependent endocytosis might be mostly exploited in 1321N1 cells, and caveolin-dependent endocytosis took a significant part in A549 cells [46]. In this study, we focused on the relation between cellular uptake and cytotoxicity of MNPs in HeLa cells. The conclusion was probably not valid for all types of cells. More effect was needed to explore the cellular uptake and cytotoxicity of MNPs in other types of cells.

In our experiment, large MNPs did not show cytotoxicity even at high concentration, but small MNPs showed obvious cytotoxicity at relative low concentration. The cytotoxicity of small MNPs could be explained by its ability to be internalized by cells. Although the reported size of MNPs collected from environment was around micrometer. For example, it was found that drinking water may contain up to 325 plastic particles with a radius of 1.5 um per liter [47]. However, we cannot exclude the risk that small MNPs existed in ambient environment and cause harm. On one hand, it has been shown that large MNPs can be transformed into small MNPs under various physicochemical effects. On another, due to the limitation of detection technology, small MNPs were difficult to be detected in real environment. The concentration of small MNPs might be much higher than expected. Considering our conclusion that small MNPs caused more harm due to their better cell uptake, a much higher healthy risk of nano-plastic particles should be expected.

Our study clarified the relation between cell uptake and cell toxicity of MNPs to HeLa cells. The key linking the two is the particle size. Although the size-dependent toxicity and the size-dependent cell uptake of MNPs has been independently studied before, the originality of our work is to build the correlation between the two. Only when we showed that the size to allow cell uptake was the same as the size to show toxicity, we could conclude that the cell uptake plays a key role in toxicity.

Since MNPs adsorbed on the cell membrane could hardly be completely removed, we cannot quantitatively measure the number of MNPs that entered the cells. Our data qualitatively showed that the entrance of MNPs into cells was determined by the particles size.

Our measurement was based on the study on HeLa cells. A series of particle size has been studied ranging from the smallest MNPs that commercially available to MNPs of micrometer. The conclusion drawn from other type of cells or other research objects, such as zebra fish, may not suitable for HeLa cells. Actually, the inconsistency of data from different research objects was already been reported. In our study the size of MNPs for cell uptake and toxicity was quantitatively measured, which would enrich the database about cytotoxicity of plastic particles.

## Conclusions

In this study, we explained the size-dependent cytotoxicity of MNPs. We found that MNPs with smaller sizes are more readily taken up by HeLa cells than larger ones. In addition, we found that MNPs that can be taken up by cells have more obvious effects on cytotoxicity than those cannot be taken up by cells. After MNPs entered the cells, they could directly touch the inner components of cells, leading to increase in ROS production and down-regulation of anti-apoptosis genes, which ultimately caused cell death. The study pointed out that the cellular uptake of MNPs is the key to its cytotoxicity, which was determined by the size of MNPs.

## Supporting information

S1 File Supporting information and raw data. (DOCX)
