# Correction: Correlation between cellular uptake and cytotoxicity of polystyrene micro/nanoplastics in HeLa cells: A size-dependent matter

**DOI:** 10.1371/journal.pone.0293935

**Published:** 2023-11-01

**Authors:** Yiming Ruan, Zheng Zhong, Xin Liu, Ziwei Li, Junxian Li, Lili Sun, Hou Sen

The order of the last author’s first and last names is switched. It should be written as: Sen Hou.
