# Correction: Correlation between cellular uptake and cytotoxicity of polystyrene micro/nanoplastics in HeLa cells: A size-dependent matter

**DOI:** 10.1371/journal.pone.0319517

**Published:** 2025-02-13

**Authors:** Yiming Ruan, Zheng Zhong, Xin Liu, Ziwei Li, Junxian Li, Lili Sun, Hou Sen

In the Funding statement, the grant number from the funder Guangdong Basic and Applied Basic Research Foundation is listed incorrectly. The correct grant number is: 2022A1515010480.
