# Supplementary material for: Correlation between cellular uptake and cytotoxicity of polystyrene micro/nanoplastics in HeLa cells: A size-dependent matter
Source: PLoS One. 2023 Aug 8;18(8):e0289473. doi: 10.1371/journal.pone.0289473 (PMC10409258; doi:10.1371/journal.pone.0289473)
Supplement: S1 File — (DOCX) [file pone.0289473.s001.docx]

Supporting Information for

**Correlation between cellular uptake and cytotoxicity of polystyrene micro/nanoplastics in HeLa cells: a size-dependent matter**

Yiming Ruan ^a^, Zheng Zhong ^a^, Xin Liu ^a^, Ziwei Li ^a^, Junxian Li ^a^, Lili Sun ^c^, Sen Hou ^a, b, d,^ *

^a^ Guangdong Key Laboratory of Environmental Pollution and Health, School of Environment, Key Laboratory of Philosophy and Social Science in Guangdong Province of Community of Life for Man and Nature, Jinan University, Guangzhou, 510632, China

^b^ CAS Key Laboratory of Soil Environment and Pollution Remediation, Institute of Soil Science, Chinese Academy of Sciences, Nanjing, 210008, China

^c^ Guangzhou Inspection Testing and Certification Group Co., Ltd., China

^d^ Shandong Huapu Testing Technology Co., Ltd. Yantai, 264000, China

* Corresponding author. E-mail address: hs0010910@jnu.edu.cn (S. Hou)

**NO. OF TABLES: 4**

**NO. OF FIGURES: 4**

**NO. OF PAGES: 21**

**Table of Contents**

Table S1. DLS parameters of polystyrene plastics in different medium

Table S2. The quality of extracted RNA.

Table S3. Primer sequences used in this study.

Figure. S1. The FTIR characterization analysis of MNPs of 10, 15, 25, 40, 50 and 500 nm in radius.

Figure S2. The cytotoxicity of background solution of MNPs.

Figure. S3. Concentration-effect curve of MNPs of (a) 10 nm in radius (b) 15 nm in radius on HeLa cell.

Figure. S4. Cytotoxicity of HeLa cells exposed to MNPs of 10 and 50 nm in radius in the same molar concentrations.

Table S4. Raw data of Figs.

**Table S1.** **Size parameters of polystyrene plastics in different medium**

| Radius (r. nm) | medium | Z-Average (r. nm) | PDI | TEM (r. nm) |
| --- | --- | --- | --- | --- |
| 10 | PBS | 12.46 ± 0.45 | 0.243 ± 0.007 | 11.00 ± 1.39 |
|  | Serum-free medium | 21.25 ± 0.77 | 0.207 ± 0.010 | - |
|  | Culture medium | 20.51 ± 1.07 | 0.787 ± 0.134 | - |
| 15 | PBS | 16.34 ± 0.17 | 0.080 ± 0.004 | 15.87 ± 0.68 |
|  | Serum-free medium | 22.52 ± 0.24 | 0.317 ± 0.010 | - |
|  | Culture medium | 27.08 ± 0.40 | 0.503 ± 0.006 | - |
| 25 | PBS | 27.20 ± 0.10 | 0.098 ± 0.010 | 25.91 ± 1.34 |
|  | Serum-free medium | 27.16 ± 0.31 | 0.182 ± 0.004 | - |
|  | Culture medium | 46.23 ± 1.94 | 0.262 ± 0.001 | - |
| 40 | PBS | 48.17 ± 2.68 | 0.140 ± 0.018 | 40.24 ± 0.78 |
|  | Serum-free medium | 50.45 ± 4.38 | 0.155 ± 0.024 | - |
|  | Culture medium | 58.45 ± 1.04 | 0.200 ± 0.002 | - |
| 50 | PBS | 56.20 ± 1.29 | 0.035 ± 0.026 | 49.93 ± 0.66 |
|  | Serum-free medium | 57.60 ± 0.82 | 0.062 ± 0.085 | - |
|  | Culture medium | 67.80 ± 0.66 | 0.178 ± 0.006 | - |
| 500 | PBS | 573.00 ± 1.41 | 0.275 ± 0.021 | 502.10 ± 4.43 |
|  | Serum-free medium | 672.50 ± 51.50 | 0.274 ± 0.012 | - |
|  | Culture medium | 936.50 ± 30.09 | 0.815 ± 0.020 | - |

**Table S2.** **The quality of extracted RNA**

| Samples | A260/A280 | A260/A230 |
| --- | --- | --- |
| Control | 1.98 | 2.06 |
| 10 nm | 1.96 | 2.12 |
| 50 nm | 1.95 | 2.03 |
| 500 nm | 1.93 | 2.14 |

**Table S3. Primer sequences used in this study**

| Gene | Primer sequence 5’-3’ |
| --- | --- |
| GAPDH | Forward Primer CATGTACGTTGCTATCCAGGC  Reverse Primer CTCCTTAATGTCACGCACGAT |
| Bax | Forward Primer CCCGAGAGGTCTTTTTCCGAG  Reverse Primer CCAGCCCATGATGGTTCTGAT |
| Bcl-2 | Forward Primer CTGAAGACTCTGCTCAGTTTG  Reverse Primer AATGATATTTCCCTTTGGCAG |
| SOD1 | Forward Primer GGTGGGCCAAAGGATGAAGAG  Reverse Primer CCACAAGCCAAACGACTTCC |
| CAT | Forward Primer TGGAGCTGGTAACCCAGTAGG  Reverse Primer CCTTTGCCTTGGAGTATTTGGTA |
| Fas | Forward Primer TCTGGTTCTTACGTCTGTTGC  Reverse Primer CTGTGCAGTCCCTAGCTTTCC |
| FADD | Forward Primer GCTGGCTCGTCAGCTCAAA  Reverse Primer ACTGTTGCGTTCTCCTTCTCT |





**Fig. S1.** The FTIR characterization analysis of MNPs of 10 nm, 15 nm, 25 nm, 40 nm, 50 nm and 500 nm in radius.





**Fig. S2.** The cytotoxicity of background solution of MNPs.


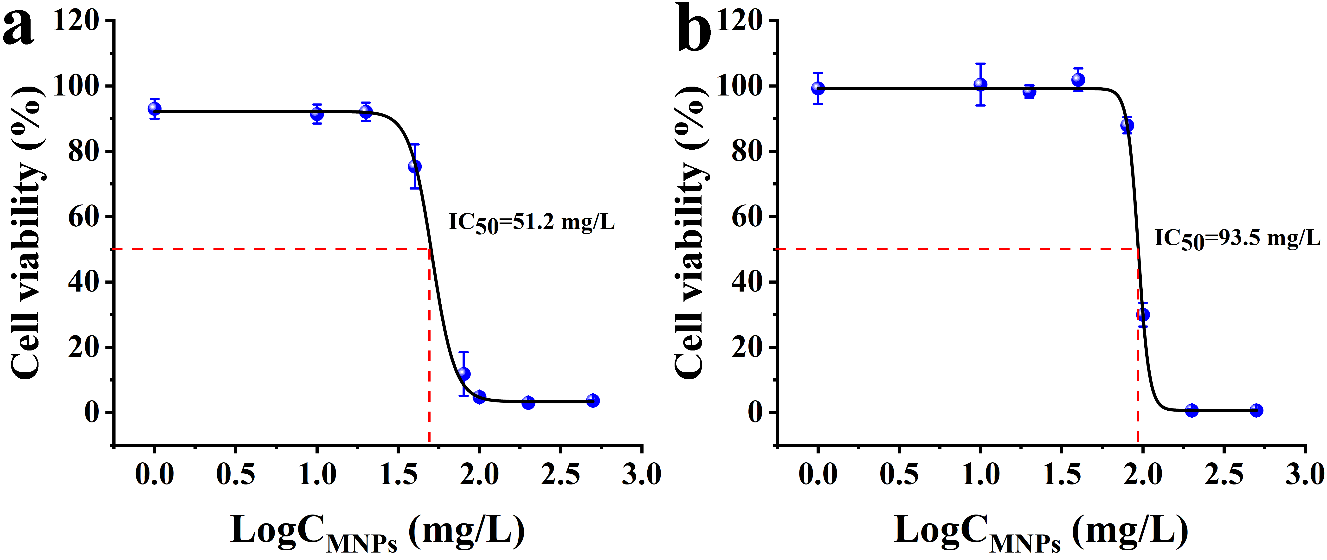


**Fig. S3**. Concentration-effect curve of MNPs of (a) 10 nm in radius (b) 15 nm in radius on HeLa cell. Cells were subjected to 4 h MNPs exposure. Results were represented with mean ± SD. The curve in Fig. S3 was fitted by the following Boltzmann function: $\text{y}\text{=}\text{y}_{\text{2}}\text{+}\frac{\text{y}_{\text{1}}\text{-}\text{y}_{\text{2}}}{\text{1+}{10}^{\text{(}\log\text{IC}_{\text{50}}\text{-x}\text{)∙}\text{Hillslope}}}$





**Fig. S4.** Cytotoxicity of HeLa cells exposed to MNPs of 10 and 50 nm in radius in the same molar concentrations.

**Table S4.** Raw data of Figs.

| PBS in Fig 1g | | | | | | |
| --- | --- | --- | --- | --- | --- | --- |
| X | 10nm | 15nm | 25nm | 40nm | 50nm | 500nm |
| 0.2 | 0 | 0 | 0 | 0 | 0 | 0 |
| 0.2315 | 0 | 0 | 0 | 0 | 0 | 0 |
| 0.268 | 0 | 0 | 0 | 0 | 0 | 0 |
| 0.3105 | 0 | 0 | 0 | 0 | 0 | 0 |
| 0.3595 | 0 | 0 | 0 | 0 | 0 | 0 |
| 0.4165 | 0 | 0 | 0 | 0 | 0 | 0 |
| 0.4825 | 0 | 0 | 0 | 0 | 0 | 0 |
| 0.56 | 0 | 0 | 0 | 0 | 0 | 0 |
| 0.645 | 0 | 0 | 0 | 0 | 0 | 0 |
| 0.75 | 0 | 0 | 0 | 0 | 0 | 0 |
| 0.87 | 0 | 0 | 0 | 0 | 0 | 0 |
| 1.005 | 0 | 0 | 0 | 0 | 0 | 0 |
| 1.165 | 0 | 0 | 0 | 0 | 0 | 0 |
| 1.35 | 0 | 0 | 0 | 0 | 0 | 0 |
| 1.56 | 0 | 0 | 0 | 0 | 0 | 0 |
| 1.81 | 0 | 0 | 0 | 0 | 0 | 0 |
| 2.095 | 0 | 0 | 0 | 0 | 0 | 0 |
| 2.425 | 0 | 0 | 0 | 0 | 0 | 0 |
| 2.805 | 0 | 0 | 0 | 0 | 0 | 0 |
| 3.25 | 0 | 0 | 0 | 0 | 0 | 0 |
| 3.765 | 0 | 0 | 0 | 0 | 0 | 0 |
| 4.36 | 0.2 | 0 | 0 | 0 | 0 | 0 |
| 5.05 | 1.3 | 0 | 0 | 0 | 0 | 0 |
| 5.85 | 3.1 | 0 | 0 | 0 | 0 | 0 |
| 6.75 | 5.5 | 0 | 0 | 0 | 0 | 0 |
| 7.85 | 7.9 | 0.1 | 0 | 0 | 0 | 0 |
| 9.1 | 10 | 2.3 | 0 | 0 | 0 | 0 |
| 10.5 | 11.5 | 6.3 | 0 | 0 | 0 | 0 |
| 12.2 | 12 | 11 | 0 | 0 | 0 | 0 |
| 14.1 | 11.5 | 14.9 | 1.2 | 0 | 0 | 0 |
| 16.35 | 10.2 | 16.9 | 4.7 | 0 | 0 | 0 |
| 18.9 | 8.2 | 16.5 | 9.4 | 0 | 0 | 0 |
| 21.9 | 5.9 | 14 | 13.8 | 0.15033 | 0 | 0 |
| 25.35 | 3.7 | 10 | 16.6 | 0.98333 | 0 | 0 |
| 29.4 | 1.8 | 5.7 | 17 | 4.71 | 0 | 0 |
| 34.05 | 0.6 | 2.2 | 15 | 10.39333 | 1.11867 | 0 |
| 39.4 | 0.1 | 0.3 | 11.3 | 15.7 | 7.82333 | 0 |
| 45.65 | 0 | 0 | 7 | 18.6 | 16.7 | 0 |
| 53 | 0 | 0 | 3.2 | 18.06667 | 22.76667 | 0 |
| 61 | 0 | 0 | 0.8 | 14.56667 | 22.86667 | 0 |
| 71 | 0 | 0 | 0 | 9.59333 | 17.26667 | 0 |
| 82 | 0 | 0 | 0 | 4.87 | 9.04333 | 0 |
| 95 | 0 | 0 | 0 | 1.81 | 2.39333 | 0 |
| 110 | 0 | 0 | 0 | 0.48 | 0 | 0 |
| 127.5 | 0 | 0 | 0 | 0.1 | 0 | 0 |
| 147.5 | 0 | 0 | 0 | 0 | 0 | 0 |
| 171 | 0 | 0 | 0 | 0 | 0 | 0 |
| 198 | 0 | 0 | 0 | 0 | 0 | 0 |
| 229.5 | 0 | 0 | 0 | 0 | 0 | 0 |
| 265.5 | 0 | 0 | 0 | 0 | 0 | 0 |
| 307.5 | 0 | 0 | 0 | 0 | 0 | 0 |
| 356 | 0 | 0 | 0 | 0 | 0 | 0 |
| 412.5 | 0 | 0 | 0 | 0 | 0 | 0 |
| 477.5 | 0 | 0 | 0 | 0 | 0 | 9.755 |
| 555 | 0 | 0 | 0 | 0 | 0 | 27.75 |
| 640 | 0 | 0 | 0 | 0 | 0 | 34 |
| 740 | 0 | 0 | 0 | 0 | 0 | 22.65 |
| 860 | 0 | 0 | 0 | 0 | 0 | 5.88 |
| 995 | 0 | 0 | 0 | 0 | 0 | 0 |
| 1150 | 0 | 0 | 0 | 0 | 0 | 0 |
| 1335 | 0 | 0 | 0 | 0 | 0 | 0 |
| 1545 | 0 | 0 | 0 | 0 | 0 | 0 |
| 1790 | 0 | 0 | 0 | 0 | 0 | 0 |
| 2075 | 0 | 0 | 0 | 0 | 0 | 0 |
| 2400 | 0 | 0 | 0 | 0 | 0 | 0 |
| 2780 | 0 | 0 | 0 | 0 | 0 | 0 |
| 3220 | 0 | 0 | 0 | 0 | 0 | 0 |
| 3730 | 0 | 0 | 0 | 0 | 0 | 0 |
| 4315 | 0 | 0 | 0 | 0 | 0 | 0 |

| DMEM in Fig1h | | | | | | |
| --- | --- | --- | --- | --- | --- | --- |
| X | 10nm | 15nm | 25nm | 40nm | 50nm | 500nm |
| 0.2 | 0 | 0 | 0 | 0 | 0 | 0 |
| 0.2315 | 0 | 0 | 0 | 0 | 0 | 0 |
| 0.268 | 0 | 0 | 0 | 0 | 0 | 0 |
| 0.3105 | 0 | 0 | 0 | 0 | 0 | 0 |
| 0.3595 | 0 | 0 | 0 | 0 | 0 | 0 |
| 0.4165 | 0 | 0 | 0 | 0 | 0 | 0 |
| 0.4825 | 0 | 0 | 0 | 0 | 0 | 0 |
| 0.56 | 0 | 0 | 0 | 0 | 0 | 0 |
| 0.645 | 0 | 0 | 0 | 0 | 0 | 0 |
| 0.75 | 0 | 0 | 0 | 0 | 0 | 0 |
| 0.87 | 0 | 0 | 0 | 0 | 0 | 0 |
| 1.005 | 0 | 0 | 0 | 0 | 0 | 0 |
| 1.165 | 0 | 0 | 0 | 0 | 0 | 0 |
| 1.35 | 0 | 0 | 0 | 0 | 0 | 0 |
| 1.56 | 0 | 0 | 0 | 0 | 0 | 0 |
| 1.81 | 0 | 0 | 0 | 0 | 0 | 0 |
| 2.095 | 0 | 0 | 0 | 0 | 0 | 0 |
| 2.425 | 0 | 0 | 0 | 0 | 0 | 0 |
| 2.805 | 0 | 0 | 0 | 0 | 0 | 0 |
| 3.25 | 0 | 0 | 0 | 0 | 0 | 0 |
| 3.765 | 0 | 0 | 0 | 0 | 0 | 0 |
| 4.36 | 0 | 0 | 0 | 0 | 0 | 0 |
| 5.05 | 0 | 0 | 0 | 0 | 0 | 0 |
| 5.85 | 0 | 0 | 0 | 0 | 0 | 0 |
| 6.75 | 1.49 | 0 | 0 | 0 | 0 | 0 |
| 7.85 | 7.61 | 1.16 | 0 | 0 | 0 | 0 |
| 9.1 | 16.4 | 5.61 | 0 | 0 | 0 | 0 |
| 10.5 | 20.6 | 12.3 | 0 | 0 | 0 | 0 |
| 12.2 | 18.1 | 17.2 | 0.17 | 0 | 0 | 0 |
| 14.1 | 12.7 | 17.9 | 2.29667 | 0 | 0 | 0 |
| 16.35 | 7.83 | 15.4 | 6.26667 | 0 | 0 | 0 |
| 18.9 | 4.66 | 11.5 | 10.76667 | 0 | 0 | 0 |
| 21.9 | 2.94 | 7.67 | 14.46667 | 0.077 | 0 | 0 |
| 25.35 | 2.02 | 4.64 | 16.2 | 1.11333 | 0 | 0 |
| 29.4 | 1.45 | 2.57 | 15.73333 | 4.37667 | 0 | 0 |
| 34.05 | 1.02 | 1.31 | 13.26667 | 9.1 | 1.11867 | 0 |
| 39.4 | 0.677 | 0.619 | 9.60333 | 13.53333 | 7.82333 | 0 |
| 45.65 | 0.438 | 0.278 | 5.69333 | 16.33333 | 16.7 | 0 |
| 53 | 0.296 | 0.132 | 2.49667 | 16.73333 | 22.76667 | 0 |
| 61 | 0.234 | 0.0807 | 0.61267 | 14.76667 | 22.86667 | 0 |
| 71 | 0.225 | 0.0735 | 0.04633 | 11.23333 | 17.26667 | 0 |
| 82 | 0.254 | 0.0879 | 0 | 7.12667 | 9.04333 | 0 |
| 95 | 0.292 | 0.111 | 0 | 3.53 | 2.39333 | 0 |
| 110 | 0.292 | 0.129 | 0 | 1.17133 | 0 | 0 |
| 127.5 | 0.238 | 0.135 | 0 | 0.20467 | 0 | 0 |
| 147.5 | 0.151 | 0.133 | 0 | 0 | 0 | 0 |
| 171 | 0 | 0.123 | 0 | 0 | 0 | 0 |
| 198 | 0 | 0.103 | 0 | 0 | 0 | 0 |
| 229.5 | 0 | 0.0739 | 0 | 0 | 0 | 0 |
| 265.5 | 0 | 0.0435 | 0 | 0 | 0 | 0 |
| 307.5 | 0 | 0.0185 | 0 | 0 | 0 | 0 |
| 356 | 0 | 0.004 | 0 | 0 | 0 | 0 |
| 412.5 | 0 | 0 | 0 | 0 | 0 | 0 |
| 477.5 | 0 | 0 | 0 | 0 | 0 | 2.15167 |
| 555 | 0 | 0 | 0 | 0 | 0 | 17.93333 |
| 640 | 0 | 0 | 0 | 0 | 0 | 31.23333 |
| 740 | 0 | 0 | 0 | 0 | 0 | 29.96667 |
| 860 | 0 | 0 | 0 | 0 | 0 | 16.13333 |
| 995 | 0 | 0 | 0 | 0 | 0 | 2.61 |
| 1150 | 0 | 0 | 0 | 0 | 0 | 0 |
| 1335 | 0 | 0 | 0 | 0 | 0 | 0 |
| 1545 | 0 | 0 | 0 | 0 | 0 | 0 |
| 1790 | 0 | 0 | 0 | 0 | 0 | 0 |
| 2075 | 0 | 0 | 0 | 0 | 0 | 0 |
| 2400 | 0 | 0 | 0 | 0 | 0 | 0 |
| 2780 | 0 | 0 | 0 | 0 | 0 | 0 |
| 3220 | 0 | 0 | 0 | 0 | 0 | 0 |
| 3730 | 0 | 0 | 0 | 0 | 0 | 0 |
| 4315 | 0 | 0 | 0 | 0 | 0 | 0 |

| DMEM contain 10 % FBS in Fig 1i | | | | | | |
| --- | --- | --- | --- | --- | --- | --- |
| X | 10nm | 15nm | 25nm | 40nm | 50nm | 500nm |
| 0.2 | 0 | 0 | 0 | 0 | 0 | 0 |
| 0.2315 | 0 | 0 | 0 | 0 | 0 | 0 |
| 0.268 | 0 | 0 | 0 | 0 | 0 | 0 |
| 0.3105 | 0 | 0 | 0 | 0 | 0 | 0 |
| 0.3595 | 0 | 0 | 0 | 0 | 0 | 0 |
| 0.4165 | 0 | 0 | 0 | 0 | 0 | 0 |
| 0.4825 | 0 | 0 | 0 | 0 | 0 | 0 |
| 0.56 | 0 | 0 | 0 | 0 | 0 | 0 |
| 0.645 | 0 | 0 | 0 | 0 | 0 | 0 |
| 0.75 | 0 | 0 | 0 | 0 | 0 | 0 |
| 0.87 | 0 | 0 | 0 | 0 | 0 | 0 |
| 1.005 | 0 | 0 | 0 | 0 | 0 | 0 |
| 1.165 | 0 | 0 | 0 | 0 | 0 | 0 |
| 1.35 | 0 | 0 | 0 | 0 | 0 | 0 |
| 1.56 | 0 | 0 | 0 | 0 | 0 | 0 |
| 1.81 | 0 | 0 | 0 | 0 | 0 | 0 |
| 2.095 | 0 | 0 | 0 | 0 | 0 | 0 |
| 2.425 | 0 | 0 | 0 | 0 | 0 | 0 |
| 2.805 | 0 | 0 | 0 | 0 | 0 | 0 |
| 3.25 | 0 | 0 | 0 | 0 | 0 | 0 |
| 3.765 | 0 | 0 | 0 | 0 | 0 | 0 |
| 4.36 | 1.1 | 0 | 0 | 0 | 0 | 0 |
| 5.05 | 1.2 | 0 | 0 | 0 | 0 | 0 |
| 5.85 | 1.4 | 0 | 0 | 0 | 0 | 0 |
| 6.75 | 2.1 | 0 | 0 | 0 | 0 | 0 |
| 7.85 | 3.1 | 0 | 0 | 0 | 0 | 0 |
| 9.1 | 4.2 | 0.1 | 0 | 0 | 0 | 0 |
| 10.5 | 5.3 | 0.4 | 0 | 0 | 0 | 0 |
| 12.2 | 6.1 | 1 | 0 | 0 | 0 | 0 |
| 14.1 | 6.5 | 2.1 | 0.09993 | 0 | 0 | 0 |
| 16.35 | 6.6 | 3.3 | 0.59667 | 0 | 0 | 0 |
| 18.9 | 6.2 | 4.4 | 1.704 | 0 | 0 | 0 |
| 21.9 | 5.5 | 5.5 | 3.44667 | 0.01143 | 0 | 0 |
| 25.35 | 4.7 | 6.4 | 5.61333 | 0.82 | 0.14633 | 0 |
| 29.4 | 3.7 | 6.9 | 7.87667 | 3.06667 | 0.83543 | 0 |
| 34.05 | 2.8 | 7.2 | 9.85333 | 6.33333 | 2.96 | 0 |
| 39.4 | 2 | 7.1 | 11.26667 | 9.67667 | 6.07333 | 0 |
| 45.65 | 1.4 | 6.9 | 11.83333 | 12.23333 | 9.3 | 0 |
| 53 | 0 | 6.4 | 11.46667 | 13.53333 | 11.84667 | 0 |
| 61 | 0 | 5.8 | 10.28667 | 13.33333 | 13.23333 | 0 |
| 71 | 0 | 5.1 | 8.43333 | 11.9 | 13.26667 | 0 |
| 82 | 0 | 4.3 | 6.23 | 9.58667 | 12.06667 | 0 |
| 95 | 0 | 3.5 | 4.02333 | 6.95333 | 10.01333 | 0 |
| 110 | 0 | 2.8 | 2.14667 | 4.44667 | 7.55333 | 0 |
| 127.5 | 0 | 2.1 | 0.84067 | 2.45333 | 5.10667 | 0 |
| 147.5 | 0 | 1.6 | 0.1827 | 1.1694 | 3.04667 | 0 |
| 171 | 0 | 1.1 | 0.009 | 0.53733 | 1.597 | 0 |
| 198 | 0 | 0.7 | 0 | 0.232 | 0.77433 | 0 |
| 229.5 | 0 | 0.4 | 0 | 0.08567 | 0.37333 | 0 |
| 265.5 | 0 | 0.2 | 0 | 0.01657 | 0.15033 | 4.82667 |
| 307.5 | 0 | 0.1 | 0 | 0 | 0.03433 | 39.76667 |
| 356 | 0 | 0 | 0 | 0 | 0 | 43.63333 |
| 412.5 | 0 | 0 | 0 | 0 | 0 | 11.75667 |
| 477.5 | 0 | 0 | 0 | 0 | 0 | 0 |
| 555 | 0 | 0 | 0 | 0 | 0 | 0 |
| 640 | 0 | 0 | 0 | 0 | 0 | 0 |
| 740 | 0 | 0 | 0 | 0 | 0 | 0 |
| 860 | 0 | 0 | 0 | 0 | 0 | 0 |
| 995 | 0 | 0 | 0 | 0 | 0 | 0 |
| 1150 | 0 | 0 | 0 | 0 | 0 | 0 |
| 1335 | 0 | 0 | 0 | 0 | 0 | 0 |
| 1545 | 0 | 0 | 0 | 0 | 0 | 0 |
| 1790 | 0 | 0 | 0 | 0 | 0 | 0 |
| 2075 | 0 | 0 | 0 | 0 | 0 | 0 |
| 2400 | 0 | 0 | 0 | 0 | 0 | 0 |
| 2780 | 0 | 0 | 0 | 0 | 0 | 0 |
| 3220 | 0 | 0 | 0 | 0 | 0 | 0 |
| 3730 | 0 | 0 | 0 | 0 | 0 | 0 |
| 4315 | 0 | 0 | 0 | 0 | 0 | 0 |

| Relative Radius in Fig 1j | | | | | | |
| --- | --- | --- | --- | --- | --- | --- |
|  | PBS | | DMEM | | DMEM cotain 10 % FBS | |
| X | Mean | SD | Mean | SD | Mean | SD |
| 10 | 1.00026 | 0.04332 | 1.67191 | 0.0847 | 1.71474 | 0.10428 |
| 15 | 0.99984 | 0.04423 | 1.3681 | 0.02027 | 1.62644 | 0.02949 |
| 25 | 0.99982 | 0.00503 | 0.9967 | 0.01248 | 1.69651 | 0.07172 |
| 40 | 0.99993 | 0.02782 | 1.18289 | 0.05579 | 1.21341 | 0.03544 |
| 50 | 1.00006 | 0.01428 | 1.091 | 0.02305 | 1.34126 | 0.12083 |
| 500 | 1 | 0.00247 | 1.17103 | 0.08516 | 1.63468 | 0.05259 |

| Cell viability in Fig 2 | | | | | | | | | | | | |
| --- | --- | --- | --- | --- | --- | --- | --- | --- | --- | --- | --- | --- |
|  | 10 nm | | 15 nm | | 25 nm | | 40 nm | | 50 nm | | 500 nm | |
| X | Mean | SD | Mean | SD | Mean | SD | Mean | SD | Mean | SD | Mean | SD |
| 0 | 1.000 | 0.070 | 1.000 | 0.045 | 1.000 | 0.059 | 1.000 | 0.037 | 1.000 | 0.031 | 1.000 | 0.009 |
| 1 | 0.930 | 0.030 | 0.993 | 0.048 | 0.997 | 0.039 | 1.007 | 0.070 | 1.012 | 0.056 | 0.953 | 0.026 |
| 10 | 0.914 | 0.029 | 1.004 | 0.064 | 0.993 | 0.030 | 1.017 | 0.070 | 1.011 | 0.007 | 1.035 | 0.005 |
| 20 | 0.921 | 0.028 | 0.983 | 0.019 | 0.978 | 0.020 | 0.990 | 0.056 | 0.972 | 0.038 | 0.935 | 0.031 |
| 40 | 0.753 | 0.068 | 1.018 | 0.035 | 1.004 | 0.019 | 0.985 | 0.035 | 1.040 | 0.106 | 0.942 | 0.011 |
| 80 | 0.118 | 0.067 | 0.879 | 0.025 | 0.982 | 0.048 | 0.974 | 0.039 | 1.057 | 0.030 | 0.960 | 0.053 |
| 100 | 0.047 | 0.005 | 0.300 | 0.036 | 0.978 | 0.041 | 0.971 | 0.043 | 1.009 | 0.061 | 0.982 | 0.051 |
| 200 | 0.030 | 0.002 | 0.006 | 0.004 | 0.969 | 0.042 | 0.963 | 0.024 | 1.030 | 0.060 | 0.979 | 0.062 |
| 500 | 0.037 | 0.001 | 0.007 | 0.003 | 0.920 | 0.024 | 0.936 | 0.019 | 1.010 | 0.049 | 0.979 | 0.015 |

| Cell death rate in Fig 3 | | | | | | | | | | | | | | | | |
| --- | --- | --- | --- | --- | --- | --- | --- | --- | --- | --- | --- | --- | --- | --- | --- | --- |
|  | | 10 nm | | | 15 nm | | | 25 nm | | | 40 nm | | 50 nm | | 500 nm | |
| X | Mean | | SD | Mean | | SD | Mean | | SD | Mean | | SD | Mean | SD | Mean | SD |
| 0 | 0.022 | | 0.008 | 0.015 | | 0.008 | 0.028 | | 0.019 | 0.030 | | 0.029 | 0.030 | 0.019 | 0.015 | 0.002 |
| 1 | 0.024 | | 0.010 | 0.020 | | 0.010 | 0.034 | | 0.023 | 0.029 | | 0.018 | 0.024 | 0.016 | 0.019 | 0.008 |
| 10 | 0.039 | | 0.012 | 0.034 | | 0.020 | 0.031 | | 0.026 | 0.048 | | 0.028 | 0.022 | 0.012 | 0.008 | 0.005 |
| 100 | 0.997 | | 0.010 | 0.789 | | 0.055 | 0.033 | | 0.019 | 0.047 | | 0.027 | 0.036 | 0.015 | 0.007 | 0.005 |
| 200 | 0.984 | | 0.011 | 0.987 | | 0.011 | 0.032 | | 0.027 | 0.022 | | 0.020 | 0.042 | 0.018 | 0.010 | 0.007 |
| 500 | 0.999 | | 0.005 | 0.983 | | 0.016 | 0.045 | | 0.012 | 0.051 | | 0.031 | 0.043 | 0.025 | 0.008 | 0.002 |

| ROS generation in Fig 6 | | | | | | | | | | | | | |
| --- | --- | --- | --- | --- | --- | --- | --- | --- | --- | --- | --- | --- | --- |
|  | 10 nm | | 15 nm | | | 25 nm | | 40 nm | | 50 nm | | 500 nm | |
| X | Mean | SD | Mean | SD | Mean | | SD | Mean | SD | Mean | SD | Mean | SD |
| 0 | 1.000 | 0.037 | 1.000 | 0.037 | 1.001 | | 0.085 | 1.001 | 0.057 | 1.000 | 0.117 | 1.001 | 0.028 |
| 1 | 1.006 | 0.077 | 1.028 | 0.075 | 1.019 | | 0.010 | 1.014 | 0.093 | 1.079 | 0.113 | 1.018 | 0.024 |
| 10 | 1.053 | 0.154 | 1.363 | 0.139 | 1.024 | | 0.030 | 1.034 | 0.094 | 1.005 | 0.160 | 0.986 | 0.032 |
| 20 | 1.390 | 0.107 | 1.508 | 0.006 | 0.948 | | 0.096 | 0.992 | 0.066 | 0.952 | 0.148 | 0.977 | 0.117 |
| 40 | 1.688 | 0.125 | 1.627 | 0.149 | 1.037 | | 0.037 | 1.045 | 0.035 | 1.033 | 0.180 | 0.985 | 0.151 |
| 80 | 1.799 | 0.116 | 1.694 | 0.055 | 1.023 | | 0.051 | 0.994 | 0.091 | 1.016 | 0.142 | 0.948 | 0.128 |
| 100 | 1.998 | 0.223 | 1.768 | 0.106 | 0.934 | | 0.072 | 0.985 | 0.066 | 1.025 | 0.142 | 0.954 | 0.045 |
| 200 | 2.049 | 0.166 | 2.115 | 0.017 | 0.975 | | 0.119 | 0.959 | 0.068 | 1.129 | 0.107 | 0.996 | 0.182 |
| 500 | 2.112 | 0.172 | 2.254 | 0.184 | 1.027 | | 0.085 | 1.027 | 0.035 | 1.037 | 0.088 | 0.929 | 0.026 |

| Gene expression levels in Fig 7 | | | | | | |
| --- | --- | --- | --- | --- | --- | --- |
|  | 10 nm | | 50 nm | | 500 nm | |
| X | Mean | SD | Mean | SD | Mean | SD |
| Bax | 0.776 | 0.225 | 0.962 | 0.308 | 1.038 | 0.144 |
| Bcl-2 | 0.765 | 0.193 | 1.009 | 0.134 | 1.022 | 0.119 |
| SOD1 | 0.867 | 0.063 | 0.923 | 0.183 | 0.974 | 0.087 |
| CAT | 0.947 | 0.057 | 0.941 | 0.038 | 0.968 | 0.193 |
| Fas | 0.749 | 0.202 | 0.978 | 0.187 | 0.977 | 0.201 |
| FADD | 0.775 | 0.252 | 0.965 | 0.263 | 0.997 | 0.108 |

| Original data for cell viability of 10 nm | | | | | | | | | |
| --- | --- | --- | --- | --- | --- | --- | --- | --- | --- |
| 0 | 1 | 10 | 20 | 40 | 80 | 100 | 200 | 500 |  |
| 0.8496 | 0.8326 | 0.8107 | 0.7940 | 0.6365 | 0.2258 | 0.2062 | 0.1908 | 0.1942 | 0.1696 |
| 0.9168 | 0.8182 | 0.8179 | 0.9102 | 0.7561 | 0.2237 | 0.1996 | 0.1909 | 0.1948 |  |
| 0.8218 | 0.7912 | 0.7801 | 0.8217 | 0.6940 | 0.3048 | 0.2015 | 0.1890 | 0.1961 |  |

| Original data for cell viability of 15 nm | | | | | | | | | |
| --- | --- | --- | --- | --- | --- | --- | --- | --- | --- |
| 0 | 1 | 10 | 20 | 40 | 80 | 100 | 200 | 500 |  |
| 1.2489 | 1.3292 | 1.2515 | 1.3014 | 1.3485 | 1.0258 | 0.4539 | 0.1581 | 0.1535 | 0.1486 |
| 1.3526 | 1.2514 | 1.377 | 1.2777 | 1.3867 | 1.1797 | 0.5373 | 0.1503 | 0.1559 |  |
| 1.295 | 1.1718 | 1.3553 | 1.257 | 1.2916 | 1.1396 | 0.4889 | 0.1593 | 0.1601 |  |

| Original data for cell viability of 25 nm | | | | | | | | |  |
| --- | --- | --- | --- | --- | --- | --- | --- | --- | --- |
| 0 | 1 | 10 | 20 | 40 | 80 | 100 | 200 | 500 |  |
| 1.0957 | 1.0491 | 1.0331 | 1.0604 | 1.0982 | 1.0638 | 0.9955 | 1.0717 | 0.9213 | 0.1711 |
| 1.0094 | 1.0465 | 1.0825 | 1.0613 | 1.1054 | 0.9211 | 1.1020 | 1.0560 | 0.9198 |  |
| 1.1066 | 1.1089 | 1.0771 | 1.0206 | 1.0133 | 0.9930 | 1.0073 | 1.0005 | 0.9704 |  |

| Original data for cell viability of 40 nm | | | | | | | | | |
| --- | --- | --- | --- | --- | --- | --- | --- | --- | --- |
| 0 | 1 | 10 | 20 | 40 | 80 | 100 | 200 | 500 |  |
| 1.2427 | 1.3206 | 1.2551 | 1.2555 | 1.2574 | 1.2679 | 0.9974 | 1.2276 | 0.9208 | 0.1652 |
| 1.2930 | 1.2775 | 1.2590 | 1.2421 | 1.2378 | 1.1917 | 1.0396 | 1.1688 | 0.9475 |  |
| 1.2339 | 1.1630 | 1.1813 | 1.2293 | 1.1702 | 1.1943 | 0.9825 | 1.1963 | 0.9043 |  |
| 1.1968 | 1.2137 | 1.3298 | 1.2056 | 1.2344 | 1.1909 | 0.9294 | 1.2154 | 0.9083 |  |

| Original data for cell viability of 50 nm | | | | | | | | | |
| --- | --- | --- | --- | --- | --- | --- | --- | --- | --- |
| 0 | 1 | 10 | 20 | 40 | 80 | 100 | 200 | 500 |  |
| 0.9328 | 0.9911 | 0.9298 | 1.0013 | 0.8512 | 1.0195 | 0.9269 | 0.9466 | 0.9260 | 0.1846 |
| 0.9651 | 0.8636 | 0.9485 | 0.8561 | 0.9907 | 0.9733 | 0.8143 | 0.8234 | 0.9895 |  |
| 0.9200 | 0.8446 | 1.0425 | 1.0114 | 0.9571 | 0.9793 | 0.9792 | 0.9526 | 0.9241 |  |

| Original data for cell viability of 500 nm | | | | | | | | | |
| --- | --- | --- | --- | --- | --- | --- | --- | --- | --- |
| 0 | 1 | 10 | 20 | 40 | 80 | 100 | 200 | 500 |  |
| 1.4753 | 1.4498 | 1.5123 | 1.0983 | 1.0915 | 1.1052 | 1.526 | 1.4409 | 1.4667 | 0.1596 |
| 1.4690 | 1.3936 | 1.5227 | 1.0786 | 1.1142 | 1.1540 | 1.41 | 1.366 | 1.4315 |  |
| 1.3153 | 1.3881 | 1.5198 | 1.1208 | 1.1004 | 1.0704 | 1.4107 | 1.5274 | 1.4346 |  |

| Original data for ROS of 10 nm | | | | | | | | |
| --- | --- | --- | --- | --- | --- | --- | --- | --- |
| 0 | 1 | 10 | 20 | 40 | 80 | 100 | 200 | 500 |
| 6273 | 7116 | 6111 | 9054 | 11118 | 12105 | 14123 | 13933 | 14663 |
| 5972 | 6043 | 6154 | 8197 | 9678 | 10939 | 11622 | 12692 | 13466 |
| 6264 | 6606 | 5997 | 8099 | 9269 | 9491 | 11804 | 10850 | 12334 |
| 6547 | 6732 | 6277 | 9485 | 10931 | 10764 | 15177 | 11873 | 12462 |

| Original data for ROS of 15 nm | | | | | | | | |
| --- | --- | --- | --- | --- | --- | --- | --- | --- |
| 0 | 1 | 10 | 20 | 40 | 80 | 100 | 200 | 500 |
| 12400 | 13419 | 15575 | 17949 | 18110 | 19676 | 22500 | 25299 | 25778 |
| 12194 | 11607 | 16356 | 17987 | 17072 | 20652 | 20839 | 25351 | 25130 |
| 11365 | 11646 | 14466 | 18130 | 20408 | 21416 | 19573 | 25641 | 28275 |
| 11982 | 11947 | 18847 | 17989 | 21183 | 20073 | 21834 | 25429 | 29361 |

| Original data for ROS of 25 nm | | | | | | | | |
| --- | --- | --- | --- | --- | --- | --- | --- | --- |
| 0 | 1 | 10 | 20 | 40 | 80 | 100 | 200 | 500 |
| 696 | 1022 | 938 | 809 | 732 | 688 | 629 | 625 | 769 |
| 664 | 920 | 916 | 729 | 762 | 754 | 724 | 789 | 766 |
| 781 | 935 | 1002 | 929 | 725 | 747 | 644 | 671 | 662 |

| Original data for ROS of 40 nm | | | | | | | | |
| --- | --- | --- | --- | --- | --- | --- | --- | --- |
| 0 | 1 | 10 | 20 | 40 | 80 | 100 | 200 | 500 |
| 876 | 873 | 882 | 917 | 1024 | 873 | 891 | 841 | 988 |
| 986 | 967 | 1050 | 1000 | 957 | 920 | 980 | 967 | 938 |
| 965 | 1049 | 1016 | 930 | 996 | 1041 | 737 | 926 | 1001 |

| Original data for ROS of 50 nm | | | | | | | | |
| --- | --- | --- | --- | --- | --- | --- | --- | --- |
| 0 | 1 | 10 | 20 | 40 | 80 | 100 | 200 | 500 |
| 878 | 880 | 794 | 709 | 975 | 841 | 831 | 1013 | 903 |
| 807 | 848 | 925 | 638 | 788 | 815 | 735 | 877 | 788 |
| 694 | 780 | 672 | 889 | 694 | 749 | 893 | 827 | 777 |

| Original data for ROS of 500 nm | | | | | | | | | | | | | | | | | | | | |  |
| --- | --- | --- | --- | --- | --- | --- | --- | --- | --- | --- | --- | --- | --- | --- | --- | --- | --- | --- | --- | --- | --- |
| 0 | | 1 | | | 10 | | 20 | | 40 | | 80 | | | 100 | | 200 | | 500 | | |  |
| 645 | | 665 | | | 618 | | 552 | | 527 | | 522 | | | 597 | | 526 | | 585 | | |  |
| 636 | | 676 | | | 646 | | 700 | | 691 | | 646 | | | 653 | | 762 | | 608 | | |  |
| 671 | | 645 | | | 659 | | 654 | | 703 | | 680 | | | 611 | | 655 | | 618 | | |  |
| Original data for gene expression levels | | | | | | | | | | | | | | | | | | | | | |
|  | 1 | | 2 | 3 | | 4 | | 5 | | 6 | | 7 | 8 | | 9 | | 10 | | 11 | 12 | |
| A | control-GAPDH | | | | | 20-GAPDH | | | | | | 30-GAPDH | | | | | 100-GAPDH | | | | |
| B | control-FAS | | | | | 20-FAS | | | | | | 30-FAS | | | | | 100-FAS | | | | |
| C | control-FADD | | | | | 20-FADD | | | | | | 30-FADD | | | | | 100-FADD | | | | |
| D | control-BAX | | | | | 20-BAX | | | | | | 30-BAX | | | | | 100-BAX | | | | |
| E | control-BCL2 | | | | | 20-BCL2 | | | | | | 30-BCL2 | | | | | 100-BCL2 | | | | |
| F | control-SOD | | | | | 20-SOD | | | | | | 30-SOD | | | | | 100-SOD | | | | |
| G | control-CAT | | | | | 20-CAT | | | | | | 30-CAT | | | | | 10-CAT | | | | |
|  |  | |  |  | |  | |  | |  | |  |  | |  | |  | |  |  | |
|  | control | | | | | 20 | | | | | | 30 | | | | | 100 nm | | | | |
|  | 1 | | 2 | 3 | | 4 | | 5 | | 6 | | 7 | 8 | | 9 | | 10 | | 11 | 12 | |
| A | 19.12 | | 19.15 | 19.10 | | 19.14 | | 19.15 | | 19.12 | | 19.07 | 19.14 | | 19.13 | | 19.12 | | 19.12 | 19.17 | |
| B | 24.14 | | 24.11 | 24.00 | | 23.88 | | 24.11 | | 24.11 | | 23.95 | 24.17 | | 24.14 | | 24.15 | | 23.99 | 23.82 | |
| C | 26.27 | | 26.48 | 26.37 | | 26.37 | | 26.06 | | 26.23 | | 26.17 | 26.13 | | 26.18 | | 26.22 | | 26.47 | 26.38 | |
| D | 25.34 | | 25.26 | 25.17 | | 24.68 | | 24.91 | | 24.73 | | 24.84 | 24.94 | | 24.70 | | 25.22 | | 25.20 | 25.45 | |
| E | 23.96 | | 23.84 | 24.01 | | 24.73 | | 24.72 | | 24.56 | | 24.64 | 24.59 | | 24.29 | | 23.85 | | 24.24 | 23.87 | |
| F | 25.01 | | 25.08 | 24.87 | | 25.45 | | 25.60 | | 25.29 | | 25.48 | 25.42 | | 25.59 | | 24.89 | | 25.21 | 24.98 | |
| G | 27.72 | | 27.85 | 27.71 | | 27.69 | | 27.74 | | 27.55 | | 27.64 | 27.77 | | 27.91 | | 27.82 | | 27.53 | 27.91 | |
